# Advances in Shellfish Allergy Therapy: From Current Approaches to Future Strategies

**DOI:** 10.1007/s12016-025-09077-8

**Published:** 2025-07-16

**Authors:** Sahel Heidari, Thimo Ruethers, Shaymaviswanathan Karnaneedi, Lydia Wong Su Yin, Andreas Ludwig Lopata

**Affiliations:** 1 Molecular Allergy Research Laboratory, Australian Institute of Tropical Health and Medicine, James Cook University, Townsville, QLD Australia; 2 Centre for Food and Allergy Research, Murdoch Children’s Research Institute, Melbourne, VIC Australia; 3 Tropical Futures Institute, James Cook University, Singapore, Singapore; 4 Khoo Teck Puat -National University Children’s Medical Institute, National University Hospital, Singapore, Singapore

**Keywords:** Shellfish allergy, Food allergy, Crustacean, Allergen-specific immunotherapy, Tropomyosin

## Abstract

Shellfish allergy, triggered by immune reactions to crustacean and mollusk proteins upon consumption/inhalation, is one of the most severe and persistent food allergies, affecting approximately 1%–3% of the general population worldwide. Shellfish is among the “big nine” food allergens responsible for over 90% of food allergy cases worldwide. Its diagnosis poses major challenges due to regional species diversity and a lack of reliable diagnostic tools. Management strategies generally emphasize strict avoidance and provision of emergency adrenaline autoinjectors; however, these approaches are inconvenient and insufficient for both patients and healthcare providers. Given the rising prevalence of shellfish allergy, there is an urgent need for targeted therapies that focus on key allergens, particularly tropomyosin—a major pan-allergen. As the primary target in current immunotherapy approaches, tropomyosin plays a central role in driving shellfish-induced immune responses. Recent advancements in immunotherapy are exploring alternatives beyond avoidance, aiming for long-term desensitization. This review discusses progress with allergen-specific immunotherapy, hypoallergenic allergen variants, DNA-based vaccines, and innovative approaches involving immunoregulatory peptides and probiotics. These strategies collectively strive to desensitize patients, reduce allergic symptoms, and enhance quality of life. Although some therapies are in active trials, most are in the investigational stages and offer promising directions for effective, patient-centered long-term management of shellfish allergy.

## Introduction

Food allergies markedly reduce quality of life and place a significant economic burden [1–3] on approximately 8% of children [4] and 11% of adults, as reported in the USA [5]. Unlike many other food allergies, shellfish and fish (seafood) allergy is rarely outgrown, and a high risk of severe allergic reactions persists throughout life, significantly impacting quality of life [5–8]. In regions such as the Asia–Pacific, where shellfish consumption is high, shellfish allergy is not only more prevalent and clinically significant, but symptoms may also develop at an earlier age compared to Western countries [9, 10]. Managing shellfish allergy is particularly challenging due to the major allergen, tropomyosin (TM), which exhibits significant cross-reactivity not only among various types of shellfish—such as crustaceans, mollusks, and cephalopods—but also with mites and other arthropods. This high degree of cross-reactivity can complicate skin testing, often necessitating multiple oral food challenges to accurately identify true clinical sensitivity. Furthermore, shellfish allergies may be specific to certain species [11, 12]. The primary approach for managing shellfish allergy remains strict avoidance [13]. Oral immunotherapy (OIT), a form of allergen-specific desensitization that increases the threshold for allergic reactions upon exposure, has been recommended by the European Academy of Allergy and Clinical Immunology (EAACI) for certain food allergies such as peanut, egg, and milk, primarily to reduce the risk of reactions from accidental exposure [13]. However, OIT is not without limitations, including adverse effects and variability in treatment outcomes depending on the criteria used to define success [14, 15]. However, it has not been developed for shellfish allergy. Most of the existing literature concentrates on peanut immunotherapy, resulting in the peanut allergen becoming the first and only treatment for food allergy approved by the United States (US) Food and Drug Administration [16]. The urgent need for effective treatments to mitigate these risks has driven advancements in immunotherapy, biologics, and other innovative therapies, paving the way for safer and more effective food allergy treatments [17]. This review will highlight key advancements in the immunotherapy of shellfish allergies.

## Overview of Shellfish Allergy

Shellfish allergy is one of the most prevalent food allergies worldwide, with region-specific high rates of severe (fatal) allergic reactions, including nearly half of affected US children experiencing life-threatening episodes [18]. The prevalence of shellfish allergy appears to range from 0.5% to 2.5% in the general population, with significant variations across different regions and age groups, and consumption habits [19, 20]. A systematic review by Moonesinghe et al. highlights a broad variation in the prevalence of shellfish allergies, ranging from less than 1% to 10.3%, depending on the method of diagnosis [21]. Unlike peanut and egg allergies, which arise in early childhood, shellfish allergy typically develops later in life [22]. In the USA, shellfish allergy affects approximately 3% (2.9%; 95% CI, 2.7%–3.1%) of adults, making it the most common food allergy among adults, affecting an estimated 7.2 million US adults [5]. In Europe, shrimp allergy prevalence rates vary, with a high of 10% in Italy and 7% in France [23], and as low as 0.3% in Denmark [24]. Additionally, shellfish allergy is a major contributor to food allergies in several regions of Asia, including Thailand, Taiwan, Hong Kong, Vietnam, and Singapore, where shellfish is a dietary staple [19, 25].

Shellfish generally refers to both groups of edible crustaceans (shrimp, crab, and lobster) and mollusks (oyster, mussels, snails, octopus, squid) [26] and is one of the “big nine” food categories that account for more than 90% of all incidents of food allergies [27, 28]. Among shellfish, shrimp is the most frequently reported allergenic species [29]. Shrimp and prawn are often used interchangeably, with prawn common in the UK and Australia, and shrimp in the USA and Europe [29]. Crustacean allergy is generally more common than mollusk allergy, with some studies reporting rates as high as 10% among Italian adults [23]. In contrast, the prevalence of mollusk allergy appears lower, particularly when confirmed through objective diagnostic methods. Nevertheless, co-allergy to both groups has been documented, with a combined prevalence of 6% among French children and 9% among American adults [21]. Similarly, a cross-sectional study in Vietnamese children reported self-reported crustacean and mollusk allergy at 3.8% and 1.3%, respectively [30]. In a physician-diagnosed adult population, seafood was identified as the predominant food allergen [31]. The increased awareness of the nutritional value of seafood has led to a surge in its consumption, reaching a global average of 20.5 kg per capita in 2020 [32] and 20.7 kg per capita in 2022 [33], according to the Food and Agriculture Organization (FAO). This increase has been associated with more frequent reports of allergic reactions. Shellfish-allergic individuals are suffering from a wide spectrum of symptoms, ranging from mild oral allergy syndrome (with limited oral symptoms of pruritus, rash, and swelling) to life-threatening anaphylaxis. Shellfish anaphylaxis stands as the leading cause of food-related fatalities in Australia, as reported by the Australian Bureau of Statistics, and has exhibited a concerning increase over the past decades [34, 35]. In Singapore [36, 37], Hong Kong [38], and Thailand [39], shellfish is the main food-related cause of anaphylaxis in adults. Shellfish allergies tend to be persistent, with low rates of resolution in the few studies available on the natural history of shellfish allergy [29, 40].

The primary allergen in shellfish is TM, a highly conserved muscle protein across species. This conservation results in considerable clinical, serological, and skin prick test (SPT) cross-reactivity among different shellfish types, complicating allergy diagnosis and management for affected individuals. Assessment of TM-specific IgE levels in shrimp-allergic individuals has shown that 72%–98% of patients have positive IgE binding to purified TM [11, 29, 41–43]. Despite being recognized as the primary allergen among shrimp-sensitized individuals, recent findings have reported sensitization rates to Pen m 1 of less than 50%, including 31% in Australian infants [44], 37% in Japanese [45], 42% in Austrian [46], 41.2% in Chinese [47], and 41% in Italian [48] shrimp-allergic populations. Additionally, studies on TM cross-reactivity with mites and insects underscore TM’s role in IgE cross-reactivity, although recent research indicates that T-cell cross-reactivity is limited and appears to depend more on protein structural stability than on amino acid sequence identity [49]. While TM is recognized as a pan-allergen, the presence of other allergens, such as arginine kinase (AK), sarcoplasmic calcium-binding protein, and hemocyanin, may influence individual clinical reactivity, as some patients may react solely to these allergens rather than the pan-allergen [46]. Identifying such novel allergens further facilitates the development of specific animal models through molecular cloning and recombinant allergen production [50–53]. Table 1 provides general information about all shrimp and crab allergens listed by the World Health Organization (WHO) and the International Union of Immunological Societies (IUIS) allergen nomenclature databases (www.allergen.org). Compared to crustaceans, knowledge of major allergens in mollusks remains limited. TM, a key allergen identified in mollusks such as abalone [54], shows a sequence identity of only 55% to 65% with TM from crustaceans, insects, mites, and fish [29]. While TM-crustacean exhibits strong immunological cross-reactivity due to a 91% conservation of IgE epitopes, mollusks display less than 20% conservation, resulting in minimal cross-reactivity between this group [55]. Additionally, we previously demonstrated in a murine model that mollusk TM can independently induce a strong IgE response, even without prior sensitization to crustacean allergens. However, this was achieved using intraperitoneal injections with alum adjuvant, which likely contributed to the observed immunogenicity [56].

**Table 1:** List of WHO-IUIS-registered shellfish allergens (www.allergen.org) on May 2025—allergic reactions have been reported after both ingestion and inhalation

	Biochemical name	Allergen (shrimp and crab)	Molecular weight	Function	Heat stable
1	Tropomyosin	Cra c 1, Mac r 1, Mel l 1, Exo m 1, Lit v 1, Met e 1, Pan b 1, Pen a 1, Pen i 1, Pen m 1	35–40 kDa	Muscle contraction	Stable
2	Arginine kinase	Cra c 2, Lit v 2, Pen m 2, Mac r 2	40–45 kDa	Energy metabolism	Labile
3	Myosin light chain 2	Lit v 3, Pen m 3	20 kDa	Muscle contraction	Stable
4	Sarcoplasmic calcium-binding protein	Cra c 4, Lit v 4, Pen m 4	20–25 kDa	Calcium ion binding	Stable
5	Myosin light chain 1	Art fr 5, Cra c 5	18 kDa	Muscle contraction	Stable
6	Troponin C	Cra c 6, Pen m 6	17–21 kDa	Muscle contraction	Unknown
7	Hemocyanin	Pen m 7	76 kDa	Oxygen transport	Labile
8	Triosephosphate isomerase	Cra c 8, Pen m 8	27–28 kDa	Glycolytic enzyme	Labile
9	Filamin C	Scy p 9	90 kDa	Cytoskeletal protein	Labile
10	Fructose bisphosphate aldolase	Cha f 10	41 kDa	Glycolysis	Unknown
11	Mitochondrial malate dehydrogenase	Para c 11	39 kDa	Citric acid cycle	Unknown
12	Cytosolic fatty acid binding protein	Lit v 13, Pen m 13	15–20 kDa	Lipid binding	Stable
13	Glycogen phosphorylase-like protein	Pen m 14	95 kDa	Glycogen breakdown	Unknown

## Diagnosis of Shellfish Allergy and Challenges

Effective diagnosis of shellfish allergy relies on obtaining a detailed medical history, including information on the type of shellfish consumed, the timing and nature of symptoms, and any treatments received [57, 58].

SPTs are commonly used as the initial diagnostic tool to assess sensitization by applying shellfish extract to the skin and monitoring for allergic reactions. While SPTs offer high sensitivity, their specificity can be limited due to cross-reactivity among shellfish species and variability in allergen content among commercial extracts [29, 59]. Moreover, some commercial SPT extracts do not contain a sufficient amount or diversity of shellfish allergens, potentially leading to false-negative results in sensitized individuals [60]. When commercial extracts are unavailable or doubtful, SPT using fresh shellfish—either raw or cooked—has been shown to improve diagnostic sensitivity, as heat treatment can affect allergen stability and IgE reactivity. However, this approach may introduce variability and requires cautious interpretation [60–62]. Serum-specific IgE testing quantifies the immune response to whole shellfish extracts or single-component allergens such as TM. Elevated IgE levels suggest sensitization, though they may not always correlate with clinical reactivity [63]. Sensitization can occur directly to shellfish allergens or through cross-reactivity with similar allergens found in other invertebrates such as cockroaches and house dust mites.

Additionally, although shellfish and finfish are taxonomically distant, limited cross-reactivity has been reported, primarily due to structurally conserved proteins like TM [29]. This may result from overlapping IgE responses, highlighting the need for cautious interpretation of test results, particularly in individuals sensitized to multiple seafood types [58, 64].

The gold standard for diagnosis is an oral food challenge (OFC), ideally a double-blind placebo-controlled food challenge (DBPCFC). However, the risk of severe allergic reactions and the time-consuming nature of these treatments limit their routine use [65]. The diagnostic process is complicated by the diverse range of shellfish species and the potential for cross-reactivity between them, necessitating the need for multiple oral food challenges. Emerging techniques such as basophil activation tests (BAT) and component-resolved diagnostics (CRD) show promise in improving diagnostic accuracy without the need for confirmatory OFC [66–69]. CRD enhances shellfish allergy assessment by identifying IgE responses to specific allergens, such as TM, which shows high specificity for shrimp allergy prediction [70]. Studies indicate that recombinant shrimp allergens, like rPen a 1, are highly effective in identifying shrimp-allergic patients [43]. CRD methods provide detailed sensitization profiles, with singleplex approaches demonstrating higher sensitivity than multiplex assays in detecting shrimp-specific IgE [71]. However, CRD still requires improvement; research has primarily focused on TM, leaving many other shellfish allergens poorly characterized. Additionally, only a limited number of shellfish species have been studied, representing just a small portion of the global variety consumed. Thus, while existing diagnostic methods for shellfish allergy offer valuable insights, ongoing research is needed to enhance their reliability and standardization, ultimately improving patient management and safety.

## Current Treatments

The most recent guideline for diagnosing and treating shellfish allergy has been established by EAACI [13]. The primary strategy for managing shellfish allergy is currently strict allergen avoidance. Individuals diagnosed with shellfish allergy are advised to eliminate all forms of shellfish from their diet, including both crustaceans and mollusks [72]. Although the majority of individuals with shrimp allergies do not exhibit respiratory symptoms from exposure to cooking steam, highly sensitive individuals may experience severe reactions to airborne allergens [73]. Therefore, it is advisable for individuals to avoid inhaling cooking fumes, steam, and vapors containing shellfish proteins, as well as refrain from touching or handling shellfish. Awareness and education play crucial roles in promoting allergen avoidance. Patients and their families should receive comprehensive information about the condition, its implications, and how to recognize allergic reactions. They should also be informed about carefully reading food labels, as shellfish can be present in various processed foods, which must be thoroughly assessed by the food industry and regulators. In addition to dietary restrictions, it is recommended to have emergency action plans and to carry personal adrenaline auto-injectors in case of severe reactions [74–76].

## Effects of House Dust Mite Immunotherapy on Shellfish Allergy

House dust mite (HDM) immunotherapy can affect shellfish allergy, highlighting the potential cross-reactivity between HDM and shellfish and the complexities in treating these overlapping sensitivities. Reports have emerged of patients who developed shrimp allergies after undergoing HDM immunotherapy, confirmed by positive SPT and food challenges. Notably, the clinical symptoms in these cases were primarily limited to oral mucosa consistent with the oral allergy hypothesis [77].

Several studies have raised concerns about potential adverse or limited effects of HDM immunotherapy for shellfish-allergic individuals. Additional insights come from studies on allergies to snail, a mollusk with cross-reactivity to HDM [78, 79]. In some cases, HDM immunotherapy has exacerbated respiratory symptoms in snail-allergic patients, even leading to anaphylaxis in individuals who previously exhibited mild symptoms [79, 80].

However, other studies have shown no significant impact of HDM immunotherapy on shellfish sensitization. For example, sublingual immunotherapy (SLIT) trials have reported no new sensitization to shrimp tropomyosin [81, 82]. One study examined the effect of immunotherapy with *Dermatophagoides pteronyssinus* extract on shrimp allergy. Among 35 patients with positive SPT, those receiving immunotherapy showed reduced skin reactivity and lower IgE levels for both mite and shrimp. After 1 year, four of ten patients with positive SPT converted to negative, and six of nine patients with shrimp-specific IgE became negative. However, no significant changes in clinical sensitivity to shrimp were observed, suggesting that mite immunotherapy may not substantially alter shrimp allergy sensitivity [83].

In contrast, some case reports and studies have highlighted potential benefits of HDM immunotherapy in improving shrimp tolerance. Evidence suggests that SLIT with HDM can increase shrimp tolerance in patients with prior anaphylaxis. This improvement was attributed to the higher dose of TM administered, as the patient received double the standard dose of SLIT [84]. Additionally, HDM SCIT has been linked to a reduction in specific serum IgE levels and, in some cases, a sustained resolution of shrimp and squid allergy symptoms, as confirmed by OFC, and maintained over a 4-year follow-up period [85]. A case report highlights a 40-year-old woman with combined allergies to mites and shrimp who underwent subcutaneous immunotherapy (SCIT) for *Dermatophagoides farinae*. After 6 months, she exhibited significant reductions in skin reactivity and serum-specific IgE levels, indicating that mite immunotherapy may aid in desensitizing patients with shrimp allergies [86]. However, the absence of a clinical challenge leaves the effect on shrimp tolerance unclear.

The opposing outcomes of HDM immunotherapy—worsening shellfish allergy in some individuals while improving it in others—highlight the need for further research to clarify the underlying pathophysiological and molecular mechanisms and to identify patient phenotypes that may benefit, before this approach can be considered a viable treatment for shellfish allergy.

## Allergen-Specific Immunotherapy Approaches

**Fig. 1:**
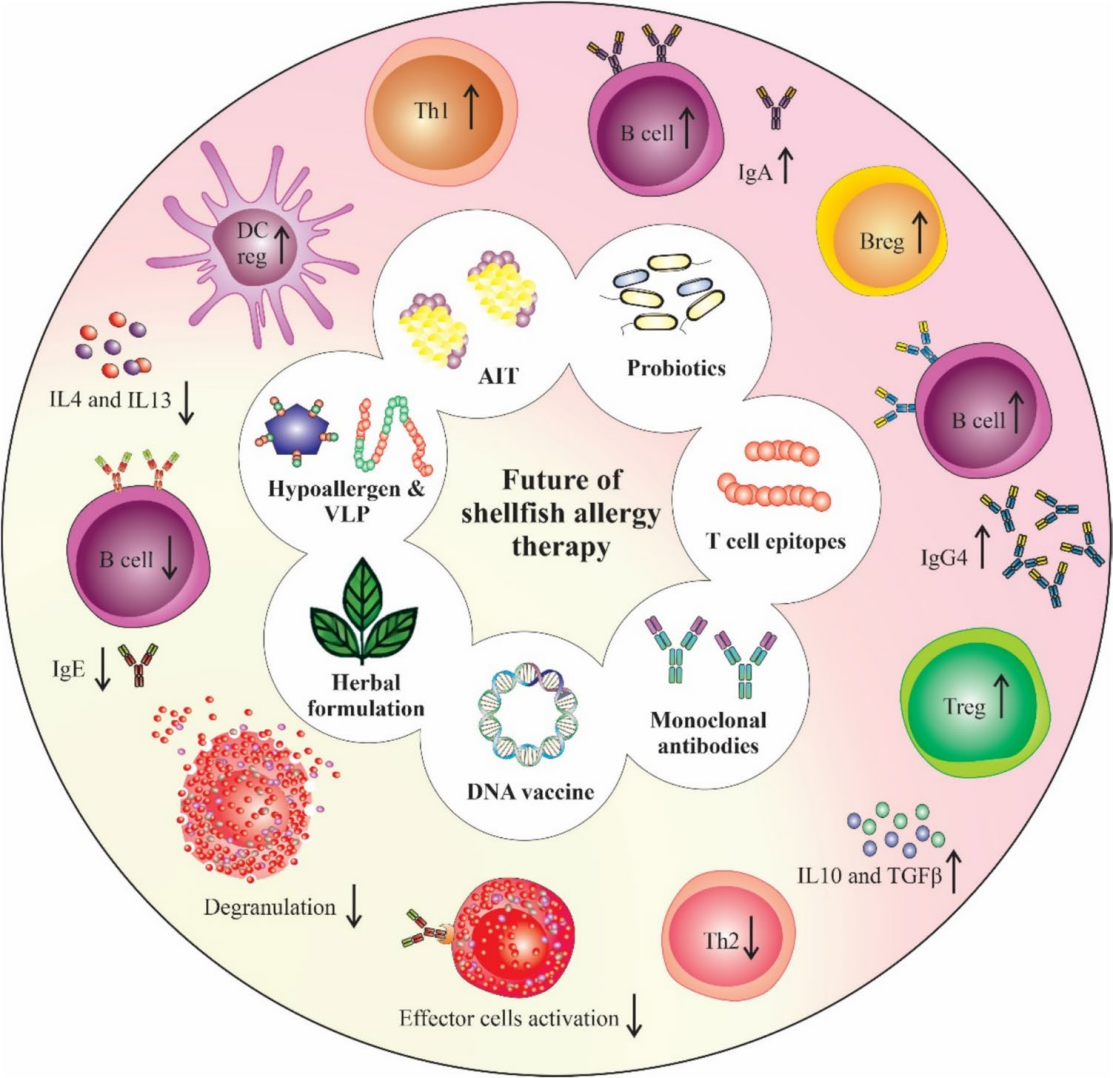
Future directions in shellfish allergy therapy. This figure illustrates various therapeutic approaches being developed to manage shellfish allergy by promoting immune tolerance and reducing allergic reactions. These strategies aim to increase regulatory cells and the production of IgG4 and IgA to minimize allergic symptoms

Shellfish allergy immunotherapy aims to induce tolerance and minimize allergic reactions through strategies such as allergen-specific immunotherapy (AIT), hypoallergenic allergen variants, monoclonal antibodies, and DNA vaccines. These methods modulate immune pathways by enhancing regulatory T cells (Treg) and regulatory B cells, increasing IgG4 and IgA, and suppressing IgE-mediated activation (Fig. 1).

### Allergen-Specific Immunotherapy

**Fig. 2:**
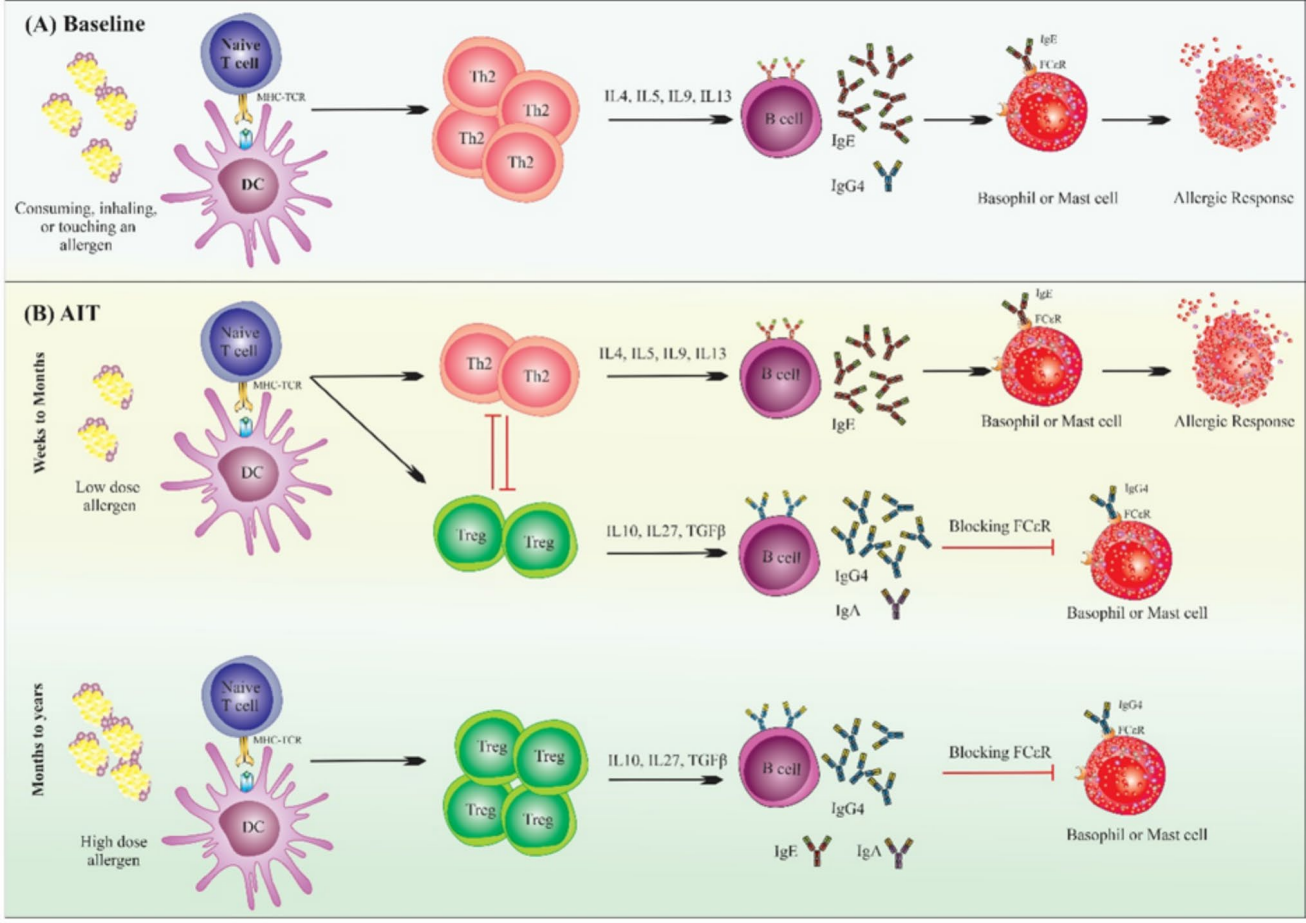
Overview of immune responses in baseline allergy and allergen-specific immunotherapy (AIT). **A** At baseline, allergen exposure activates dendritic cells (DCs), which prime naive T cells towards a Th2 response. Th2 cells release cytokines (IL-4, IL-5, IL-9, IL-13), inducing IgE production by B cells. IgE binds Fcε receptors on mast cells and basophils, triggering degranulation and allergic reactions upon re-exposure. **B** During AIT, repetitive low-dose allergen exposure over weeks to months induces a shift towards Tregs that suppress Th2 responses and increase IL-10, IL-27, and TGF-β production. B cells switch from IgE to IgG4 production, with IgG4 functioning as a blocking antibody that prevents IgE from binding Fcε receptors, thereby inhibiting mast cell and basophil degranulation. Prolonged high-dose allergen exposure over months to years further enhances Treg responses and stabilizes IgG4-mediated blocking, resulting in long-term tolerance and reduced allergic responses

The concept of AIT originates in the early twentieth century and remained largely unexplored for several decades due to the high risk of severe reactions and anaphylaxis. However, recent advancements offer promising safe possibilities for achieving desensitization and long-term management of food allergies [87, 88]. AIT involves regular exposure to increasing doses of the allergen—in this case, shellfish proteins—to induce desensitization and tolerance. Initially, allergen-specific IgE levels may rise, but they typically decline below baseline over time. The maintenance phase generally requires daily consumption of a target dose of the allergen, aiming to raise the threshold of reactivity and reduce the severity of allergic reactions in cases of accidental exposure. While the exact mechanism remains under investigation, current evidence suggests that AIT first suppresses basophils and mast cells, followed by a shift towards the formation of allergen-specific Tregs, the depletion of reactive mediators, and the stimulation of allergen-specific IgG production. Over time, Th2 cell activity diminishes, while regulatory cells, such as Tregs and Bregs that produce IL-10, become more prominent [87, 88]. Allergen-specific IgG, particularly IgG4, is believed to act as a “blocking antibody” by binding to the allergen before it interacts with IgE on effector cells, thereby preventing mast cell and basophil activation (Fig. 2).

Various administration routes and dosing strategies are considered in AIT for food allergies, including OIT, SLIT, SCIT, and epicutaneous immunotherapy (EPIT) [89, 90]. Among these, OIT has shown potential as a treatment for food allergies such as peanut and milk; however, concerns about its safety and tolerability remain major obstacles to its wider application [91, 92]. In comparison, SLIT offers a safer option with fewer side effects, though it is generally less effective [92, 93]. Currently, no clinical data currently exists for the use of SCIT or EPIT in shellfish immunotherapy. This review focuses on recent advancements in OIT and SLIT for shellfish allergy.

#### Oral Immunotherapy

Despite the high prevalence of shellfish allergy, studies investigating shellfish OIT remain limited in scope and species (only shrimp). OIT has shown promise as a treatment for food allergies, including shellfish, but its application is constrained by safety concerns. While most reactions during OIT are mild, severe reactions can occur, particularly during the initial and buildup phase. Chronic symptoms, such as abdominal pain potentially linked to eosinophilic esophagitis (EoE), are a common reason for discontinuation of therapy [91]. Recent advancements, such as combining OIT with biologics such as omalizumab, aim to enhance both safety and efficacy [94]. Evidence indicates that initiating treatment early, particularly in patients with high IgE levels, may enhance the likelihood of achieving long-term desensitization and remission [95]. Although new protocols have been developed to improve safety, further research is necessary to refine OIT and maximize its effectiveness for managing shellfish allergies.

Nguyen et al. examined the effectiveness of OIT for shrimp-allergic patients in a multi-food OIT trial that included omalizumab as an adjuvant [96]. This case series, which involved three patients—two of whom were children under 18—provided initial evidence suggesting that shrimp OIT may be a viable strategy for managing shrimp allergies, despite the small sample size. In another recent study, a novel shrimp OIT approach was explored, which focused on bypassing the traditional build-up phase and proceeding directly to a maintenance dose [97]. This case series involved 17 mild shrimp-allergic or shrimp-sensitized pediatric patients, who were administered a maintenance dose of 300 mg shrimp protein (equivalent to 1.6 g of cooked shrimp) without the initial low-dose build-up phase. The study hypothesized that due to shrimp’s higher reaction threshold compared to other allergenic foods, this approach could be safe for patients with mild shrimp allergies. A Phase 2 trial assessed the safety and efficacy of OIT for cashew and shrimp allergies by administering a 1000 mg maintenance dose over 52 weeks [98]. The trial demonstrated that OIT safely desensitized individuals with mild to moderate adverse reactions, primarily gastrointestinal symptoms. Mechanistic analyses showed significant increases in allergen-specific IgG4 and alterations in allergen-reactive CD4 + T cells, indicating effective desensitization.

New shrimp allergy models are being developed to improve the understanding of allergic mechanisms and advance diagnostic and therapeutic tools for shellfish allergy [99–101]. In a mouse model of gastrointestinal allergies, the potential of Pacific white prawn (*Litopenaeus vannamei*) as an allergen extract for immunotherapy was explored [102]. Mice were divided into groups and treated with varying doses—high, moderate, and low—of the shrimp extract, undergoing sensitization, desensitization, and subsequent oral challenges. Shrimp allergen extract (SAE) immunotherapy reduced systemic allergic symptoms across all dosages, with persistent effects after multiple challenges. Notably, high-dose treatment significantly increased IgG2a levels and IL-10 mRNA expression, highlighting a dose-dependent immunotherapy effect.

The lack of standardized allergen thresholds often creates confusion and risky decisions for individuals with food allergies. Without sufficient data on minimum doses unlikely to trigger reactions, developing effective allergen management strategies becomes challenging for both clinicians and patients [103].

A key consideration in OIT is the threshold at which allergic reactions are triggered. Previous studies have suggested that shrimp exhibits a higher reaction threshold compared to other allergenic foods, likely due to its relatively lower allergenic protein content (9,10). Houben et al. demonstrated that mustard is the most potent allergenic food, whereas soy and shrimp have the lowest allergenic potential [104], making shrimp allergies unique in the context of OIT protocols.

To further investigate this threshold, DBPCFCs were conducted with shrimp-allergic adults. Participants received increasing doses of shrimp mixed in a seasoned beef matrix, ranging from 100 µg to 4 g. The study revealed that the most sensitive individuals reacted at 2.5 mg of shrimp protein. Additionally, the estimated dose predicted to provoke reactions in 5% of the shrimp-allergic population (ED05) was with 73.6–127 mg higher than those for other common food allergens, such as peanut, milk, and egg. These findings suggest that shrimp-allergic individuals generally have a higher reaction threshold, though further challenges are needed to confirm these results [105].

The importance of dose and threshold was also explored in a BALB/c murine model for shrimp hypersensitivity, where varying doses of a recombinant shrimp tropomyosin (rMet e 1) were administered to assess allergic responses and immune changes. While all treated mice were desensitized and protected during subsequent challenges, high-dose treatment caused severe systemic reactions. However, low and medium doses led to the upregulation of Treg-associated genes and an increase in Foxp3 + cells in gut tissues, suggesting that low-dose immunotherapy promotes local regulatory T cell induction and regulatory cytokine upregulation. This finding highlights the potential safety and long-term efficacy of low-dose immunotherapy in managing shrimp allergy [106].

#### Sublingual Immunotherapy

SLIT is another promising approach that involves placing allergen extracts under the tongue, allowing for absorption through the oral mucosa. It has emerged as a safer alternative to OIT for food allergy, offering a less invasive and lower-risk approach, albeit at the cost of reduced efficacy. A study conducted on 60 patients (aged from 5 to 50 years) with shrimp allergies evaluated the safety and efficacy of SLIT [107, 108]. Participants were divided into groups based on symptoms such as urticaria, rhinitis, and asthma and were treated with shrimp extract from two shrimp species (*Penaeus semisulcatus* and *Metapenaeus stebbingi*) administered sublingually. After 6 months of treatment, there was a significant reduction in allergic symptoms, accompanied by a decrease in specific IgE levels and an increase in IgG4 levels. While the study provides valuable insights, certain methodological aspects require careful consideration, such as the uncommon presentation of isolated rhinitis and the absence of oral food challenges to confirm outcomes. Future studies with comprehensive clinical assessments should further validate these findings.

A study conducted in a Midwest Allergy-Immunology practice included 66 patients with shrimp allergies, consisting of both children and adults, mainly presenting with systemic or localized reactions to shrimp. Patients were treated with serially diluted shrimp extracts, starting at doses of 64–320 ng and gradually increasing to 0.5 mg/dose three times a day over a treatment duration ranging from 5 to 72 months. A subset of patients (18/66) underwent challenges, of whom 61% tolerated 42 g or more (~ 4 shrimp) of shrimp. While some localized reactions occurred, no severe adverse events were reported, underscoring that SLIT could be a safe and effective desensitization approach for shrimp allergy in select patients [109].

### Hypoallergen-Based Immunotherapy

Oral desensitization has proven to be an effective strategy for achieving immune tolerance in individuals with food allergies. However, despite its effectiveness, conventional approaches using native allergens frequently lead to allergic reactions, underscoring the need for safer alternatives. One such approach involves the use of hypoallergenic allergens, which was first trialled for fish allergy [110]. Hypoallergens are designed to maintain immunogenicity while minimizing the risk of triggering allergic reactions, thus enhancing both the safety and efficacy of food allergy immunotherapy [111]. Modifying allergenic epitopes—regions of the protein that bind to IgE antibodies—has become a key area of focus for researchers and the food industry [112]. In shellfish allergies, much of this work has centered on modifying TM, the primary allergen in many shellfish species [63].

#### Effect of Digestion and Food Processing on Allergenicity

Epitopes can be classified into two types: linear and conformational [113]. Linear epitopes are continuous amino acid sequences, while conformational epitopes are composed of discontinuous sequences that fold together through the protein’s three-dimensional structure [114]. It has been reported that most IgE epitopes are conformational; however, food processing and digestion often disrupt these structures, leaving linear epitopes more likely to reach the immune system in a reactive state. Nonetheless, under certain conditions, conformational epitopes may remain intact despite processing and digestion [115, 116].

Although digestion primarily influences the natural allergenicity of food proteins by altering their structural integrity, understanding how digestion affects these allergenic epitopes can provide valuable insights for designing hypoallergenic variants for immunotherapy. During digestion, proteins are broken down into smaller fragments, which can alter both conformational and linear epitopes that are crucial for immune recognition [117, 118]. It has been shown that even peptides, despite often being unstable during digestion, may still induce sensitization. Structural changes, such as protein unfolding and aggregation, can either disrupt existing epitopes or create new ones, influencing the protein’s sensitivity or resistance to gastric and gastrointestinal enzymes [119]. For example, a study on shrimp (*Penaeus vannamei*) demonstrated that while gastric digestion reduces allergic reactions, gastrointestinal digestion can actually increase the allergenic potential of shrimp proteins, likely due to the masking or exposure of specific epitopes during digestion [120]. TM from mud crab (*Scylla serrata*) demonstrated high resistance to digestion in simulated gastric and intestinal fluids, unlike other proteins like myosin heavy chain and actin, which were rapidly degraded [121]. Similarly, TM from shrimp (*Penaeus vannamei*) and Chinese mitten crab (*Eriocheir sinensis*) showed resistance to digestive enzymes, although partial reductions in allergenicity were observed. Their digestion-resistant fragments retained allergic activity, highlighting anti-digestion as a critical factor in allergenicity [122, 123].

The processing of food allergens is another factor that influences allergenicity, as thermal treatments and gastrointestinal digestion often destroy conformational epitopes. However, linear epitopes can persist, making them a key factor in the allergenicity of shrimp, even after processing [124]. Some shellfish allergens, such as TM and sarcoplasmic calcium-binding protein from *Litopenaeus vannamei*, retained its IgE and IgG-binding capacities even after undergoing various cooking methods [125]. One study found that roasting shrimp (*Penaeus vannamei*) increased allergenicity by exposing more linear epitopes. However, when roasting was combined with reverse-pressure sterilization, allergenic responses were significantly reduced. This combination masked stable epitopes within protein aggregates and enhanced the gastrointestinal digestion of immunodominant epitopes. Mice treated with this combined method exhibited a weaker anaphylactic response, lower levels of specific antibodies, and reduced signs of cell degranulation compared to those treated with only roasted or reverse-pressure sterilized proteins [124]. Yadzir et al. investigated the effects of boiling, frying, and roasting on oyster allergenicity and found that thermal treatment generally reduced allergenicity by decreasing the number of IgE-reactive bands. Interestingly, allergenicity was highest in raw extracts, followed by boiled, with fried and roasted extracts showing similar levels of allergenicity [126]. More recently, it was demonstrated that boiling shrimp can increase IgE reactivity, likely due to the preservation of digestion-resistant allergenic fragments [127]. Consistently, research on TM in shrimp and oysters revealed that cooking does not significantly reduce allergenic risk, as TM remains intact after heat treatment, leading to higher IgE reactivity in roasted extracts compared to raw forms [128, 129]. Also, thermal processing affects T-cell reactivity, as cooked extracts increase IgE reactivity and reduce Treg levels compared to raw extracts, highlighting the immune-modulatory effects of cooking [130].

Among thermal processing methods, high-pressure steaming has emerged as a particularly effective way to reduce the allergenicity of TM in *Penaeus monodon*, surpassing the effectiveness of other heat treatments [131]. Gamma radiation combined with heat significantly reduced shrimp allergen immunoreactivity, with higher radiation doses showing greater effects [132]. High-intensity ultrasound at 50 °C significantly reduced the major shrimp allergen Pen a 1 and IgE binding, while no effect was observed at 0 °C [133]. This study evaluated the effects of three processing methods—boiling, combined ultrasound and boiling, and high-pressure steaming—on TM from crab. High-pressure steaming was the most effective in reducing TM’s IgE/IgG reactivity and enhancing its degradation during gastrointestinal digestion, suggesting it as a promising method to lower crab allergenicity [134].

#### Glycation and Allergen Modification

Another promising approach to reduce allergenicity involves the glycation of shrimp TM. Glycation, the process of bonding a protein with a sugar molecule, has been shown to reduce the allergenicity of TM in several studies: In a study using a mast cell degranulation system and a murine model, glycated TM with smaller saccharides led to significantly lower histamine release, reduced IgE levels, and milder allergic symptoms. When combined with Al(OH)₃, glycated TM also promoted a shift towards regulatory and Th1 responses and milder anaphylactic symptoms after mice OFC, supporting its potential as a candidate for shrimp allergy immunotherapy. The study also showed that saccharide size was critical, with smaller saccharides producing greater glycation and allergenicity reduction, while TM glycated with maltose had no significant effect [135]. Further research demonstrated that glycation of shrimp TM from species such as *Penaeus aztecus* [136], *Litopenaeus vannamei* [137], *Exopalaemon modestus* [138], and *Penaeus chinensis* [139] with various saccharides also shows potential as a method for developing hypoallergenic candidates for immunotherapy.

A meta-analysis on OIT found that processing methods, including Maillard-treated allergens and slightly processed crustacean meat, significantly reduced anaphylactic symptoms in mice and improved oral tolerance in clinical patients, supporting their potential role in OIT strategies [140].

#### Enzyme Treatments

In addition to glycation, enzymatic hydrolysis is a promising method for producing hypoallergenic shrimp products. Enzyme treatments, such as transglutaminase (TG) and tyrosinase, have been explored for their ability to reduce the allergenicity of TM by altering its structure and reducing its IgE-inducing capacity. Furthermore, TG-treated TM has been shown to promote Treg proliferation, contributing to its hypoallergenic properties and potential use in food production and immunotherapy [141]. The use of papain, a common enzyme in the food industry, was shown to reduce the TM allergenicity in shrimp [142]. The study found that treating shrimp meat with 20U of papain, combined with 3 min of heating, decreased TM levels by up to 80%. Additionally, Fourier-transform infrared spectroscopy analysis revealed alterations in the secondary protein structure, highlighting this processing method’s potential to produce hypoallergenic shrimp products. In another approach, hypoallergenic variants of Cra g 1 were developed through epitope deletion and site-directed mutagenesis [143]. These variants showed significantly reduced IgE reactivity, degranulation, and allergic mediator secretion, suggesting their potential for use in clinical immunotherapy for shellfish allergies.

#### Innovative Strategies for Hypoallergens Under Investigation

Beyond these biological modifications, advancements in immunotherapy have extended to the development of novel delivery systems for hypoallergens. Recently, AP205-based virus-like particles (VLPs) were created using the SpyTag/SpyCatcher system combined with the Pen m 1 allergen. These VLPs have shown reduced allergenicity while enhancing the production of TM-specific IgG-blocking antibodies, further improving the safety profile of immunotherapy. Future studies are required to evaluate the efficacy of this method in preventing shrimp-induced anaphylaxis in animal models and eventually in human clinical trials [144, 145].

Efforts to create hypoallergenic variants of shrimp TM have also made significant progress. Research on shrimp TM, Met e 1, has identified specific IgE-binding epitopes and led to the development of two hypoallergenic variants, MEM49 and MED171. These variants exhibited significantly reduced IgE reactivity and allergenicity while inducing IgG antibodies that blocked IgE binding, making them promising candidates for shrimp allergy immunotherapy [146]. Epitope mapping studies on shrimp TM fragments revealed that the N- and C-terminal regions exhibit strong IgE-binding and receptor crosslinking, highlighting key allergenic domains that could inform hypoallergenic design strategies [147]. An evaluation of five Pen a 1 epitopes demonstrated that epitope 3 plays a crucial role in allergenicity, while epitope 5 remained stable across all treatment conditions, including irradiation and heat treatment [148]. Furthermore, Li et al. have shown that hypoallergenic derivatives of mud crab (*Scylla paramamosain*) allergens, specifically through the elimination of dominant linear epitopes in Scy p 1 and Scy p 3, could be promising candidates for immunotherapy [116]. The Pen a 1 mutant VR9-1, carrying 12 amino acid substitutions across major IgE-binding epitopes, demonstrated a remarkable 90%–98% reduction in allergenic potency, suggesting its potential as a therapeutic agent in shellfish immunotherapy [149]. These derivatives, including mutant allergens with deleted heat- and digestion-stable linear epitopes, were unable to bind to IgE or induce basophil activation in some patients. Additionally, Chen et al. identified the allergen AK in *Oratosquilla oratoria* and developed an epitope-deleted derivative, mAK-L, which demonstrated reduced immunoreactivity compared to recombinant AK [150].

### DNA Vaccine-Based Immunotherapy

DNA vaccines can trigger TH1-dominant immune responses, which makes them suitable for allergen-specific therapies. This TH1 bias can be further amplified by co-expressing TH1 cytokines alongside the vaccine antigens. IL-12, a robust TH1-inducing cytokine, has shown significant potential as a DNA vaccine adjuvant in both small and large animal studies and has proven to be safe and effective in humans [151–153]. This innovative strategy involves the administration of plasmid DNA encoding specific shellfish allergens, intending to modulate the immune response and induce tolerance.

Wai et al. introduced two shrimp hypoallergens, MEM49 and MED171, and evaluated their effectiveness as DNA vaccines in reducing shellfish allergy symptoms in mice. The intradermal administration of pMED171 resulted in a significant reduction of allergic responses, primarily through the induction of Treg, which are crucial for maintaining immune tolerance. This treatment notably decreased anaphylactic symptoms and intestinal inflammation following oral allergen challenges [154]. Another study utilized a DNA plasmid vaccine encoding shrimp antigens and a lysosomal-associated membrane protein, demonstrating its ability to suppress anaphylactic reactions by inducing a strong Th1 response characterized by increased levels of IgG2a, IL-10, and IFN-γ [155].

One of the most promising aspects of DNA vaccines is their ability to induce the production of allergen-specific IgG antibodies. These IgG antibodies play a dual role: (a) they can intercept allergens before they bind to cell surface-bound IgE or (b) engage inhibitory receptors like FcγRIIb on effector cells, thus mitigating the allergic response. DNA vaccines delivered via Gene Gun targeting shellfish allergens led to a significant increase in shrimp-specific IgG production across several mouse strains, with C3H/HeJ mice showing the highest response. Importantly, the vaccine also induced IgG responses against lobster and crab allergens, indicating its potential for broader cross-reactivity in crustacean allergies [152].

To enhance the efficacy of DNA vaccines, co-delivery of immunomodulatory molecules such as IL-12 has been explored. This method aims to further skew the immune response towards a TH1 phenotype, reducing the TH2-driven allergic response [156]. While DNA vaccines show great promise in preclinical studies, challenges remain in optimizing delivery methods and ensuring long-term efficacy, particularly when translating findings from animal models to human clinical trials. Ongoing research is required to refine DNA vaccine strategies, addressing these issues to improve the safety and effectiveness of shellfish allergy treatments. Translating preclinical findings into effective patient treatments will necessitate further advancements in delivery methods and long-term efficacy.

### Peptide-Based Immunotherapy

Peptide-based immunotherapy (PIT) is an approach gaining considerable attention for allergic diseases. This method uses short synthetic peptides containing allergen-specific CD4 + T cell epitopes, which induce tolerance by stimulating Tregs and promoting a Th1 response [157]. These peptides have a significantly reduced capacity to crosslink IgE, and consequently, they do not activate mast cells or basophils, reducing the risk of allergic reactions [158, 159]. Recent research on TMs, including Pen m 1, demonstrated that T-cell cross-reactivity is influenced more by structural stability than by sequence similarity [49], providing insights for PIT development for shrimp allergies.

A key area of interest in PIT is to identify allergen-specific epitopes for different allergens. For instance, Ravkov et al. identified 17 epitopes from shrimp TM and validated them as capable of inducing T cell proliferation and cytokine release (IL-6 and IL-13) in shrimp-allergic individuals. These epitopes, restricted to common MHC class II alleles, are ideal candidates for PIT [160]. Similarly, Wai et al. evaluated immunodominant T cell epitopes of TM from *Metapenaeus ensis* (Met e 1) in a Balb/c mouse model. Mice treated with the peptide mixture exhibited reduced allergic symptoms, including a significant decrease in Th2-related antibodies and cytokines [161]. Furthermore, PIT using the T-cell epitope of AK encapsulated with the TLR9 agonist CpG-ODN in nanoparticles, demonstrated significant attenuation of Th2-mediated allergic responses, reducing anaphylactic symptoms and Th2 cytokines while enhancing Th1 cytokine expression in a shrimp allergy model [162].

Beyond synthetic peptides, another approach involves mimotopes—short peptides that mimic allergenic epitopes—which have been investigated for targeting shrimp allergens. A study used the one-bead-one-compound library to identify multiple mimotopes that bind TM-specific IgE. These mimotopes were validated through peptide ELISA, epitope mapping, and immunization in a Balb/c mouse model, demonstrating their ability to induce TM-specific IgG without triggering allergic reactions [163].

### Monoclonal Antibody-Based Immunotherapy

Anti-IgE therapy has been under investigation for many years and has recently shown promising results in the management of IgE-mediated food allergy. Omalizumab, a humanized monoclonal antibody targeting free IgE, has been evaluated for its ability to reduce allergic reactions following accidental food exposure [164]. Clinical trials have demonstrated that it can increase the tolerated dose of various food allergens (including peanut, milk, and egg) both as monotherapy and in combination with OIT [165]. Beyond omalizumab, other anti-IgE monoclonal antibodies such as talizumab and ligelizumab have also been investigated. Talizumab showed dose-dependent increases in reactivity thresholds in early peanut allergy trials but was not further developed [166]. Ligelizumab, a next-generation anti-IgE with higher affinity for IgE, has shown potential in early studies; however, it has not yet demonstrated superior clinical benefit to omalizumab in the context of food allergy [164, 167, 168]. To date, omalizumab remains the most extensively studied and the only anti-IgE biologic with Food and Drug Administration (FDA) approval for food allergy [164, 169].

However, data specific to shellfish allergy remain scarce. One pilot study involving 22 patients with asthma and concomitant IgE-mediated food allergy reported reduced reactions to various foods, including shellfish, after six doses of omalizumab, with improvement observed in symptoms such as atopic dermatitis, urticaria, rhinosinusitis, and anaphylaxis [170]. Additionally, a recruiting clinical trial (NCT06369467) is evaluating linvoseltamab—a novel anti-IgE monoclonal antibody—in combination with dupilumab in adults with severe IgE-mediated food allergies, including shellfish, offering a potential avenue for future targeted therapies.

## Adjuvant/Complementary Therapies

### Probiotics

Probiotics, defined as live microorganisms that confer health benefits to the host when administered in adequate amounts, have been increasingly studied for their role in regulating both the immune system and gut microbiota [171–173]. These microorganisms have demonstrated significant potential in providing preventive and therapeutic benefits for allergic conditions, including shellfish allergies [174]. Schiavi et al. investigated the effects of the VSL#3 probiotic mixture in a shrimp TM-induced mouse model. The results showed that VSL#3 reduced allergic reactions, such as anaphylaxis and histamine release, by shifting the immune response from a Th2-dominated profile to a more balanced Th1/T regulatory profile. This shift decreased pro-inflammatory cytokines like IL-4, IL-5, and IL-13, while increasing anti-inflammatory cytokines such as IL-10 and TGF-β [175]. Similarly, Fu et al. highlighted the potential of yogurt-sourced probiotic bacteria, *Bifidobacterium longum* and *Bacillus coagulans*, in mitigating shrimp TM-induced allergic responses in a BALB/c mouse model by restoring gut microbiota balance and regulating immune responses [176]. In another shrimp allergy model, *Bifidobacterium infantis* was shown to increase Tregs and balance Th2/Treg ratios, suggesting that probiotics may play a valuable role in managing shellfish allergies through immune modulation [177].

Additionally, oxidative stress has been identified as a factor in the sensitization to allergens. Probiotics, which contain antioxidant enzymes such as glutathione peroxidase and superoxide dismutase, may help reduce oxidative stress by scavenging reactive oxygen species and regulating DCs. This modulation, in conjunction with immune regulation, positions probiotics as a promising therapeutic approach for shellfish allergies [178]. Studies indicate that children with shrimp or crab sensitization have significantly lower GPx activity, further linking oxidative stress to allergic responses [179].

### Traditional Chinese Medicine

Research on traditional Chinese medicine (TCM) for treating food allergies remains relatively rare [180]. However, TCM is being explored as a therapeutic option for allergic diseases, including food allergies. Li et al.’s [181] study on a combination of 11 herbs in a mouse model of peanut allergy demonstrated promising results. Ongoing clinical trials are exploring the therapeutic use of Chinese herbal formulations for various food allergies, with encouraging findings reported for several types, including shellfish allergy [182]. The clinical investigation of Food Allergy Herbal Formula-2 (FAHF-2) began after its approval as an investigational new drug by the US FDA in 2007, based on successful murine studies. A phase I trial involving 18 participants with peanut, tree nut, fish, and shellfish allergies demonstrated that FAHF-2 was well tolerated and showed significant immune-modulating effects, including reduced IL-5 and increased IFN‐γ. In the phase II trial, extended over 6 months, FAHF-2 continued to show long-term tolerability and BAT reduction. The main outcome was the change in reaction threshold during OFCs before and after treatment. Although the treatment was well tolerated and in vitro studies showed that FAHF-2 suppressed IL-5, induced IL-10, and increased Tregs, indicating a shift to a non-allergic immune response, the primary endpoint was not achieved, possibly due to poor adherence by 44% of participants [182–184]. In another study using a murine model of multiple food allergies, FAHF-2 also protected against allergen-induced anaphylaxis in multiple food allergies (peanut, fish, and egg) [180, 185].

## Clinical Trials

Multiple clinical trials have investigated potential immunotherapy treatments for shellfish allergies, as summarized in Table 2. This table includes 10 trials on shellfish immunotherapy, encompassing OIT, Chinese herbal formulas, monoclonal antibodies, and alternative techniques. Key findings suggest that OIT is generally safe, with mild to moderate adverse events, and shows promise for desensitization and increased IgG4 levels. Chinese herbal formulas, such as FAHF-2, demonstrated favorable in vitro effects but require further clinical validation. Monoclonal antibodies such as linvoseltamab and omalizumab are being studied for their potential to enhance safety and efficacy. In contrast, alternative approaches like Nambudripad’s Allergy Elimination Techniques—a nonconventional method combining acupressure and muscle testing—have been proposed but lack scientific validation or robust clinical evidence [186]. These studies demonstrate progress in shellfish immunotherapy but underscore the need for continued research to establish long-term safety and efficacy.

**Table 2:** Summary of clinical trials of shellfish immunotherapy listed by ClinicalTrials.gov (www.clinicaltrials.gov)

Study details	NCT	Phase	Status	Condition	Intervention type	Sample size	Age (years)	Purpose	Location/duration	Findings
Immunotherapy approaches										
Short-Term Linvoseltamab Treatment on Top of Chronic Dupilumab Treatment for Adults With Severe IgE-Mediated Food Allergy	NCT06369467	1	Recruiting	Food allergy (including shellfish)	Linvoseltamab and dupilumab	6	18–50	Evaluate the effects of Linvoseltamab in patients undergoing Dupilumab treatment for severe food allergy	USA (2024–2025 Estimated)	Not submitted
Open-label Extension Study of ADP101	NCT05243719	1 and 2	Completed	Food allergy (including shellfish)	ADP101 OIT	45	4–57	Evaluate the safety and efficacy of ADP101 for OIT	USA (2022–2024)	Not submitted
Multi OIT to Test Immune Markers After Minimum Maintenance Dose	NCT03181009	2	Completed	Food allergy (multiple allergens)	Omalizumab and multi-Food OIT	60	2–25	Explore if Omalizumab can enhance safety and enable lower maintenance doses in OIT	USA (2017–2019)	−70% showed changes sIgG4/sIgE ratio -Early plasma marker changes occur even at a 300 mg dose with mOIT and omalizumab [187]
Randomized Double-Blind Placebo-Controlled Crab or Shrimp Allergy Reduction Study Using Nambudripad Allergy Elimination Techniques	NCT02208414	N/A	Unknown	Crab or shrimp allergy	NAET^1^	80	> 20	Evaluate NAET with acupressure or chiropractic techniques over 6 months for shellfish	Taiwan (2012–2014)	Not submitted
ADP101 for Oral Immunotherapy in Food-Allergic Children and Adults	NCT04856865	1 and 2	Completed	Food allergy (including shellfish)	ADP101 OIT	73	4–55	Evaluate the safety and efficacy of ADP101 for food allergy	USA (2021–2022)	Advisor and Patient Advisory Committee feedback improved ADP101 palatability, refined trial materials, and ensured timely enrollment, with plans to expand patient input in phase 3 [188]
Food Allergen OIT for Shrimp and Cashew	NCT03504774	2	Completed	Shrimp and cashew nut allergy	OIT	52	7–55	Assess desensitization and sustained unresponsiveness to shrimp and cashew allergens	USA (2019–2023)	-Shrimp OIT was safe, with mainly mild (90%) and some moderate (10%) adverse events -Increased sIgG4 [98]
Immunological Response After Shrimp Oral Immunotherapy Treatment	NCT04552522	N/A	Not yet recruiting	Shrimp allergy	OIT	20	12–40	Investigate immune responses (sIgE and IgG4) and tolerance post-oral immunotherapy	Thailand (2020–2024)	Not submitted
Jak Inhibition in Food Allergy	NCT05069831	1	Recruiting	Food allergy (including shellfish)	Abrocitinib (JAK Inhibitor)	40	18–50	Examine the potential of abrocitinib, a JAK1 inhibitor, to reduce food allergy symptoms by measuring BAT, SPT, and sIgE changes over 4 months	USA (2022–2024)	Not submitted
Adjuvant/complementary approaches										
Therapeutic Effect of Chinese Herbal Medicine on Food Allergy	NCT00602160	2	Unknown	Food allergy (including shellfish)	FAHF-2 herbal formula	68	12–45	Assess FAHF-2 for food allergy, targeting multiple allergens	USA (2007–2012)	-FAHF-2 was safe and well-tolerated, with favorable in vitro effects (e.g., reduced IL-5, increased IL-10, Tregs) -Placebo showed a higher eliciting dose in DBPCFC, with no differences in IgE/IgG4 or epinephrine use [182]
Pilot study on traditional Chinese medicine and food allergy	NCT02490813	N/A	Completed	Allergy to cod, shrimp, crab	Chinese herbal formula-X	18	> 8	Assess allergic tolerance after 8-week treatment with Chinese herbal formula-X	Hong Kong (2015–2023)	Not submitted

^1^NAET, Nambudripad Allergy Elimination Techniques

## Future Directions

The future of shellfish immunotherapy will likely be shaped by advancements in precision diagnostics and personalized treatment strategies, aimed at enhancing both safety and efficacy for patients with severe allergies. Shellfish OIT is a promising option; however, several key questions remain unsolved, including optimal dosing, the duration of maintenance, and how to optimize treatment for patients who are allergic to multiple species of shellfish. Currently, most allergen-specific immunotherapy research for shrimp and shellfish allergy are largely confined to animal models and proof-of-concept studies, limiting their progression to clinical trials and practical application in clinical settings. A major challenge in advancing these therapies is the absence of standardized protocols and comprehensive safety data, both of which are critical for ensuring the reproducibility, scalability, and clinical effectiveness of treatments in human patients.

Given the significant cross-reactivity observed between crustaceans and mollusks, modifying key allergens, such as TM, may offer the potential for broader desensitization across various shellfish species. However, further research is essential to fully elucidate the structural and molecular characteristics of major shellfish allergens. This includes identifying specific T- and B-cell epitopes and analyzing the interactions of IgE with their allergen binding sites. Precision medicine approaches, such as diagnostic tests that assess an individual’s specific allergen recognition profile, could enhance the understanding of shellfish allergies and improve the efficacy of therapeutic interventions.

Another key challenge is the considerable diversity of shellfish allergens. As noted, most allergen-specific immunotherapy strategies focus on TM, despite its variable prevalence across different geographical regions, where it is not always the dominant allergen. This variability complicates the development of universal immunotherapy approaches, as they must account for regional and individual differences in allergen sensitization patterns.

Advances in allergen modification, such as the development of hypoallergenic variants through epitope deletion or structural alterations, hold promise for inducing tolerance without triggering severe allergic reactions. The combination of immunotherapy with biologic agents, including anti-IgE, anti-IL4, and anti-IL13 antibodies, may further enhance both safety and efficacy, particularly in patients with multiple allergies. Emerging strategies, such as microbiome modulation and targeted delivery systems, also show significant potential for reshaping immune responses. Altering the microbiome through interventions such as probiotics may help promote tolerance, while novel approaches such as virus-like particles or DNA vaccines could offer targeted, long-term desensitization with minimal side effects.

## Conclusion

Shellfish allergy continues to present ongoing challenges due to its severity, lifelong persistence, and the diversity of allergenic proteins across various species. Although strict avoidance remains the primary management strategy, emerging immunotherapeutic approaches such as allergen-specific immunotherapy, hypoallergenic variants, DNA vaccines, and microbiome-based interventions are reshaping future treatment paradigms. These innovations hold promise for inducing long-term tolerance, but their clinical application is limited by regional variability, insufficient standardization, and a lack of human trials. Advancing shellfish immunotherapy will require the integration of precision diagnostics, targeted interventions, and robust clinical validation to ensure safe, effective, and individualized care.

## Data Availability

No datasets were generated or analysed during the current study.

## References

[1] Morou Z, Vassilopoulou E, Galanis P, Tatsioni A, Papadopoulos NG, Dimoliatis IDK (2021) Investigation of quality of life determinants in children with food allergies. Int Arch Allergy Immunol 182(11):1058–1065. 10.1159/000516875 34192693 10.1159/000516875

[2] Patel GB, Kellner ES, Clayton E, Chhiba KD, Alakija O, Bryce PJ et al (2021) Quality of life is lower in adults labeled with childhood-onset food allergy than in those with adult-onset food allergy. Ann Allergy Asthma Immunol 127(1):70–75. 10.1016/j.anai.2021.03.009 33753218 10.1016/j.anai.2021.03.009 PMC8483056

[3] Blaiss MS, Meadows JA, Yu S, Robison DR, Hass SL, Norrett KE et al (2021) Economic burden of peanut allergy in pediatric patients with evidence of reactions to peanuts in the United States. J Manag Care Spec Pharm 27(4):516–527. 10.18553/jmcp.2021.20389 33470880 10.18553/jmcp.2021.20389 PMC10394212

[4] Gupta RS, Warren CM, Smith BM et al (2018) The public health impact of parent-reported childhood food allergies in the United States. Pediatrics 142(6):e20181235. 10.1542/peds.2018-3835 30455345 10.1542/peds.2018-1235 PMC6317772

[5] Gupta RS, Warren CM, Smith BM, Jiang J, Blumenstock JA, Davis MM et al (2019) Prevalence and severity of food allergies among US adults. JAMA Netw Open 2(1):e185630. 10.1001/jamanetworkopen.2018.5630 30646188 10.1001/jamanetworkopen.2018.5630 PMC6324316

[6] Sicherer SH, Munoz-Furlong A, Sampson HA (2004) Prevalence of seafood allergy in the United States determined by a random telephone survey. J Allergy Clin Immunol 114(1):159–165. 10.1016/j.jaci.2004.04.018 15241360 10.1016/j.jaci.2004.04.018

[7] Boyce JA, Assa’ad A, Burks AW, Jones SM, Sampson HA, Panel NI-SE et al (2010) Guidelines for the diagnosis and management of food allergy in the United States: report of the NIAID-sponsored expert panel. J Allergy Clin Immunol. 126(6 Suppl):S1-58. 10.1016/j.jaci.2010.10.007 21134576 10.1016/j.jaci.2010.10.007 PMC4241964

[8] Loo EXL, Lau HX, Suaini NHA, Wong LSY, Goh AEN, Teoh OH et al (2021) House dust mite sensitization, eczema, and wheeze increase risk of shellfish sensitization. Pediatr Allergy Immunol 32(5):1096–1099. 10.1111/pai.13493 33687761 10.1111/pai.13493 PMC7611115

[9] Lee AJ, Gerez I, Shek LP, Lee BW (2012) Shellfish allergy–an Asia-Pacific perspective. Asian Pac J Allergy Immunol 30(1):3–10 22523902

[10] Klaewsongkram J (2012) High prevalence of shellfish and house dust mite allergies in Asia-Pacific: probably not just a coincidence. Asian Pac J Allergy Immunol 30(4):247–248 23393903

[11] Kamath SD, Bublin M, Kitamura K, Matsui T, Ito K, Lopata AL (2023) Cross-reactive epitopes and their role in food allergy. J Allergy Clin Immunol 151(5):1178–1190. 10.1016/j.jaci.2022.12.827 36932025 10.1016/j.jaci.2022.12.827

[12] Wong L, Huang CH, Lee BW (2016) Shellfish and house dust mite allergies: is the link tropomyosin? Allergy Asthma Immunol Res 8(2):101–106. 10.4168/aair.2016.8.2.101 26739402 10.4168/aair.2016.8.2.101 PMC4713872

[13] Santos AF, Riggioni C, Agache I, Akdis CA, Akdis M, Alvarez-Perea A et al (2024) EAACI guidelines on the management of IgE-mediated food allergy. Allergy. 10.1111/all.16345 39473345 10.1111/all.16345 PMC11724237

[14] Berkes S, Liddell K, Beyer K, Blumchen K, Deschildre A, Kukkonen K et al (2025) Re-evaluating treatment success in trials of peanut oral-immunotherapy: impact of different definitions on efficacy outcomes. Curr Opin Allergy Clin Immunol 25(3):185–193. 10.1097/ACI.0000000000001077 40233247 10.1097/ACI.0000000000001077 PMC12052049

[15] Loke P (2025) The conversation on oral immunotherapy for preschool children must continue. Clin Exp Allergy 55(4):288–290. 10.1111/cea.70027 40015931 10.1111/cea.70027

[16] Fernandez-Rivas M, Vereda A, Vickery BP, Sharma V, Nilsson C, Muraro A et al (2022) Open-label follow-on study evaluating the efficacy, safety, and quality of life with extended daily oral immunotherapy in children with peanut allergy. Allergy 77(3):991–1003. 10.1111/all.15027 34320250 10.1111/all.15027 PMC9293305

[17] Sindher SB, Long A, Chin AR, Hy A, Sampath V, Nadeau KC et al (2022) Food allergy, mechanisms, diagnosis and treatment: innovation through a multi-targeted approach. Allergy 77(10):2937–2948. 10.1111/all.15418 35730331 10.1111/all.15418

[18] Lau CH, Springston EE, Smith B, Pongracic J, Holl JL, Gupta RS (2012) Parent report of childhood shellfish allergy in the United States. Allergy Asthma Proc 33(6):474–480. 10.2500/aap.2012.33.3610 23394504 10.2500/aap.2012.33.3610

[19] Mederos-Luis E, Poza-Guedes P, Pineda F, Sanchez-Machin I, Gonzalez-Perez R (2024) Gastropod allergy: a comprehensive narrative review. Curr Issues Mol Biol 46(6):5950–5964. 10.3390/cimb46060355 38921026 10.3390/cimb46060355 PMC11202862

[20] Woo CK, Bahna SL (2011) Not all shellfish “allergy” is allergy! Clin Transl Allergy 1(1):3. 10.1186/2045-7022-1-3 22410209 10.1186/2045-7022-1-3 PMC3294628

[21] Moonesinghe H, Mackenzie H, Venter C, Kilburn S, Turner P, Weir K et al (2016) Prevalence of fish and shellfish allergy: a systematic review. Ann Allergy Asthma Immunol 117(3):264–72 e4. 10.1016/j.anai.2016.07.015 27613460 10.1016/j.anai.2016.07.015

[22] Kamdar TA, Peterson S, Lau CH, Saltoun CA, Gupta RS, Bryce PJ (2015) Prevalence and characteristics of adult-onset food allergy. J Allergy Clin Immunol Pract 3(1):114–115. 10.1016/j.jaip.2014.07.007 25577631 10.1016/j.jaip.2014.07.007 PMC4578642

[23] Burney P, Summers C, Chinn S, Hooper R, van Ree R, Lidholm J (2010) Prevalence and distribution of sensitization to foods in the European Community Respiratory Health Survey: a EuroPrevall analysis. Allergy 65(9):1182–1188. 10.1111/j.1398-9995.2010.02346.x 20180791 10.1111/j.1398-9995.2010.02346.x

[24] Osterballe M, Hansen TK, Mortz CG, Host A, Bindslev-Jensen C (2005) The prevalence of food hypersensitivity in an unselected population of children and adults. Pediatr Allergy Immunol 16(7):567–573. 10.1111/j.1399-3038.2005.00251.x 16238581 10.1111/j.1399-3038.2005.00251.x

[25] Wai CYY, Leung NYH, Leung ASY, Wong GWK, Leung TF (2021) Seafood allergy in Asia: geographical specificity and beyond. Front Allergy 2:676903. 10.3389/falgy.2021.676903 35387013 10.3389/falgy.2021.676903 PMC8974776

[26] Lopata AL, O’Hehir RE, Lehrer SB (2010) Shellfish allergy. Clin Exp Allergy 40(6):850–858. 10.1111/j.1365-2222.2010.03513.x 20412131 10.1111/j.1365-2222.2010.03513.x

[27] Adedeji AA, Priyesh PV, Odugbemi AA (2024) The magnitude and impact of food allergens and the potential of AI-based non-destructive testing methods in their detection and quantification. Foods. 13(7):994. 10.3390/foods13070994 38611300 10.3390/foods13070994 PMC11011628

[28] Spolidoro GCI, Ali MM, Amera YT, Nyassi S, Lisik D, Ioannidou A et al (2023) Prevalence estimates of eight big food allergies in Europe: updated systematic review and meta-analysis. Allergy 78(9):2361–2417. 10.1111/all.15801 37405695 10.1111/all.15801

[29] Ruethers T, Taki AC, Johnston EB, Nugraha R, Le TTK, Kalic T et al (2018) Seafood allergy: a comprehensive review of fish and shellfish allergens. Mol Immunol 100:28–57. 10.1016/j.molimm.2018.04.008 29858102 10.1016/j.molimm.2018.04.008

[30] Le TTK, Nguyen DH, Vu ATL, Ruethers T, Taki AC, Lopata AL (2019) A cross-sectional, population-based study on the prevalence of food allergies among children in two different socio-economic regions of Vietnam. Pediatr Allergy Immunol 30(3):348–355. 10.1111/pai.13022 30793379 10.1111/pai.13022

[31] Le TTK, Tran TTB, Ho HTM, Vu ATL, McBryde E, Lopata AL (2020) The predominance of seafood allergy in Vietnamese adults: results from the first population-based questionnaire survey. World Allergy Organ J 13(3):100102. 10.1016/j.waojou.2020.100102 32161634 10.1016/j.waojou.2020.100102 PMC7058921

[32] Tacon AGJ, Shumway SE (2024) Critical need to increase aquatic food production and food supply from aquaculture and capture fisheries: trends and outlook. Rev Fish Sci Aquacult 32:389–395

[33] Food and Agriculture Organization of the United Nations (FAO) (2024) The state of world fisheries and aquaculture 2024: blue transformation in action. Rome: FAO, p 215 10.4060/cd0683en

[34] Mullins RJ, Wainstein BK, Barnes EH, Liew WK, Campbell DE (2016) Increases in anaphylaxis fatalities in Australia from 1997 to 2013. Clin Exp Allergy 46(8):1099–1110. 10.1111/cea.12748 27144664 10.1111/cea.12748

[35] Koeberl M, Clarke D, Allen KJ, Fleming F, Katzer L, Lee NA et al (2018) Food allergen management in Australia. J AOAC Int 101(1):60–69. 10.5740/jaoacint.17-0386 29202903 10.5740/jaoacint.17-0386

[36] Thong BY, Cheng YK, Leong KP, Tang CY, Chng HH (2007) Immediate food hypersensitivity among adults attending a clinical immunology/allergy centre in Singapore. Singapore Med J 48(3):236–240 17342294

[37] Liew WK, Chiang WC, Goh AE, Lim HH, Chay OM, Chang S et al (2013) Paediatric anaphylaxis in a Singaporean children cohort: changing food allergy triggers over time. Asia Pac Allergy 3(1):29–34. 10.5415/apallergy.2013.3.1.29 23403810 10.5415/apallergy.2013.3.1.29 PMC3563018

[38] Smit DV, Cameron PA, Rainer TH (2005) Anaphylaxis presentations to an emergency department in Hong Kong: incidence and predictors of biphasic reactions. J Emerg Med 28(4):381–388. 10.1016/j.jemermed.2004.11.028 15837017 10.1016/j.jemermed.2004.11.028

[39] Lertnawapan R, Maek-a-nantawat W (2011) Anaphylaxis and biphasic phase in Thailand: 4-year observation. Allergol Int 60(3):283–289. 10.2332/allergolint.10-OA-0256 21364308 10.2332/allergolint.10-OA-0256

[40] Zotova V, Clarke AE, Chan ES, Asai Y, Chin R, Van Lambalgen C et al (2019) Low resolution rates of seafood allergy. J Allergy Clin Immunol Pract 7(2):690–692. 10.1016/j.jaip.2018.09.011 30253936 10.1016/j.jaip.2018.09.011

[41] Ayuso R, Sanchez-Garcia S, Lin J, Fu Z, Ibanez MD, Carrillo T et al (2010) Greater epitope recognition of shrimp allergens by children than by adults suggests that shrimp sensitization decreases with age. J Allergy Clin Immunol 125(6):1286–1293. 10.1016/j.jaci.2010.03.010 20471069 10.1016/j.jaci.2010.03.010

[42] Bauermeister K, Wangorsch A, Garoffo LP, Reuter A, Conti A, Taylor SL et al (2011) Generation of a comprehensive panel of crustacean allergens from the North Sea Shrimp Crangon crangon. Mol Immunol 48(15–16):1983–1992. 10.1016/j.molimm.2011.06.216 21784530 10.1016/j.molimm.2011.06.216

[43] Gamez C, Sanchez-Garcia S, Ibanez MD, Lopez R, Aguado E, Lopez E et al (2011) Tropomyosin IgE-positive results are a good predictor of shrimp allergy. Allergy 66(10):1375–1383. 10.1111/j.1398-9995.2011.02663.x 21651567 10.1111/j.1398-9995.2011.02663.x

[44] Kamath SD, Johnston EB, Iyer S, Schaeffer PM, Koplin J, Allen K et al (2017) IgE reactivity to shrimp allergens in infants and their cross-reactivity to house dust mite. Pediatr Allergy Immunol 28(7):703–707. 10.1111/pai.12764 28782222 10.1111/pai.12764

[45] Tsedendorj O, Chinuki Y, Ueda K, Kohno K, Adachi A, Morita E (2018) Tropomyosin is a minor but distinct allergen in patients with shrimp allergies in Japan. J Cutan Immunol Allergy 1(3):100–108. 10.1002/cia2.12019

[46] Grilo J, Vollmann U, Aumayr M, Sturm GJ, Bohle B (2022) Tropomyosin is no accurate marker allergen for diagnosis of shrimp allergy in Central Europe. Allergy 77(6):1921–1923. 10.1111/all.15290 35322447 10.1111/all.15290 PMC9321988

[47] Wai CYY, Leung NYH, Leung ASY, Ngai SM, Pacharn P, Yau YS et al (2022) Comprehending the allergen repertoire of shrimp for precision molecular diagnosis of shrimp allergy. Allergy 77(10):3041–3051. 10.1111/all.15370 35567339 10.1111/all.15370 PMC9795902

[48] Asero R, Mistrello G, Amato S, Ariano R, Colombo G, Conte ME et al (2012) Shrimp allergy in Italian adults: a multicenter study showing a high prevalence of sensitivity to novel high molecular weight allergens. Int Arch Allergy Immunol 157(1):3–10. 10.1159/000324470 21894023 10.1159/000324470

[49] Kamath SD, Scheiblhofer S, Johnson CM, Machado Y, McLean T, Taki AC et al (2020) Effect of structural stability on endolysosomal degradation and T-cell reactivity of major shrimp allergen tropomyosin. Allergy 75(11):2909–2919. 10.1111/all.14410 32436591 10.1111/all.14410 PMC7687109

[50] Parvataneni S, Gonipeta B, Acharya HG, Gangur V (2015) An adjuvant-free mouse model of transdermal sensitization and oral elicitation of anaphylaxis to shellfish. Int Arch Allergy Immunol 168(4):269–276. 10.1159/000443736 26895004 10.1159/000443736

[51] Smeekens JM, Kesselring JR, Bagley K, Kulis MD (2024) A mouse model of shrimp allergy with cross-reactivity to crab and lobster. Methods Mol Biol 2717:311–319. 10.1007/978-1-0716-3453-0_21 37737994 10.1007/978-1-0716-3453-0_21 PMC11328323

[52] Leung PS, Lee YS, Tang CY, Kung WY, Chuang YH, Chiang BL et al (2008) Induction of shrimp tropomyosin-specific hypersensitivity in mice. Int Arch Allergy Immunol 147(4):305–314. 10.1159/000144038 18617750 10.1159/000144038

[53] Liu T, Navarro S, Lopata AL (2016) Current advances of murine models for food allergy. Mol Immunol 70:104–117. 10.1016/j.molimm.2015.11.011 26759987 10.1016/j.molimm.2015.11.011

[54] Lopata AL, Zinn C, Potter PC (1997) Characteristics of hypersensitivity reactions and identification of a unique 49 kd IgE-binding protein (Hal-m-1) in abalone (Haliotis midae). J Allergy Clin Immunol 100(5):642–648. 10.1016/s0091-6749(97)70168-4 9389294 10.1016/s0091-6749(97)70168-4

[55] Dramburg S, Hilger C, Santos AF, de Las Vecillas L, Aalberse RC, Acevedo N et al (2023) EAACI Molecular Allergology User’s Guide 2.0. Pediatr Allergy Immunol 34 Suppl 28:e13854. 10.1111/pai.13854 10.1111/pai.13854 37186333

[56] Kamath SD, Liu T, Giacomin P, Loukas A, Navarro S, Lopata AL (2022) Mollusk allergy: not simply cross-reactivity with crustacean allergens. Allergy 77(10):3127–3130. 10.1111/all.15377 35575977 10.1111/all.15377 PMC9796110

[57] Wai CYY, Leung PSC (2022) Emerging approaches in the diagnosis and therapy in shellfish allergy. Curr Opin Allergy Clin Immunol 22(3):202–212. 10.1097/ACI.0000000000000827 35660713 10.1097/ACI.0000000000000827

[58] Tong WS, Yuen AW, Wai CY, Leung NY, Chu KH, Leung PS (2018) Diagnosis of fish and shellfish allergies. J Asthma Allergy 11:247–260. 10.2147/JAA.S142476 30323632 10.2147/JAA.S142476 PMC6181092

[59] Asero R, Scala E, Villalta D, Pravettoni V, Arena A, Billeri L et al (2017) Shrimp allergy: analysis of commercially available extracts for in vivo diagnosis. J Investig Allergol Clin Immunol 27(3):175–182. 10.18176/jiaci.0127 27959286 10.18176/jiaci.0127

[60] Ruethers T, Johnston EB, Karnaneedi S, Nie S, Nugraha R, Taki AC et al (2023) Commercial shellfish skin prick test extracts show critical variability in allergen repertoire. Allergy 78(12):3261–3265. 10.1111/all.15853 37602511 10.1111/all.15853 PMC10952831

[61] Tsabouri S, Triga M, Makris M, Kalogeromitros D, Church MK, Priftis KN (2012) Fish and shellfish allergy in children: review of a persistent food allergy. Pediatr Allergy Immunol 23(7):608–615. 10.1111/j.1399-3038.2012.01275.x 22554093 10.1111/j.1399-3038.2012.01275.x

[62] Rance F, Juchet A, Bremont F, Dutau G (1997) Correlations between skin prick tests using commercial extracts and fresh foods, specific IgE, and food challenges. Allergy 52(10):1031–1035. 10.1111/j.1398-9995.1997.tb02427.x 9360758 10.1111/j.1398-9995.1997.tb02427.x

[63] Lopata AL, Kleine-Tebbe J, Kamath SD (2016) Allergens and molecular diagnostics of shellfish allergy: Part 22 of the Series Molecular Allergology. Allergo J Int 25(7):210–218. 10.1007/s40629-016-0124-2 28239537 10.1007/s40629-016-0124-2 PMC5306157

[64] Lv L, Wei F, Liu L, Song F, Hou X, Yang Q (2025) Study on the allergenicity of tropomyosin from different aquatic products based on conformational and linear epitopes analysis. J Agric Food Chem 73(8):4936–4946. 10.1021/acs.jafc.4c11853 39948035 10.1021/acs.jafc.4c11853

[65] Rubin ZE, Gu H, Polk BI (2020) Seafood graded oral food challenge outcomes in a pediatric tertiary care center. World Allergy Organ J 13(5):100121. 10.1016/j.waojou.2020.100121 32477447 10.1016/j.waojou.2020.100121 PMC7248448

[66] Santos AF, Alpan O, Hoffmann HJ (2021) Basophil activation test: mechanisms and considerations for use in clinical trials and clinical practice. Allergy 76(8):2420–2432. 10.1111/all.14747 33475181 10.1111/all.14747

[67] Borres MP, Maruyama N, Sato S, Ebisawa M (2016) Recent advances in component resolved diagnosis in food allergy. Allergol Int 65(4):378–387. 10.1016/j.alit.2016.07.002 27543004 10.1016/j.alit.2016.07.002

[68] Pascal M, Grishina G, Yang AC, Sanchez-Garcia S, Lin J, Towle D et al (2015) Molecular diagnosis of shrimp allergy: efficiency of several allergens to predict clinical reactivity. J Allergy Clin Immunol Pract 3(4):521–9 e10. 10.1016/j.jaip.2015.02.001 25769902 10.1016/j.jaip.2015.02.001

[69] Wai CYY, Leung NYH, Leung ASY, Shum Y, Leung PSC, Chu KH et al (2021) Cell-based functional IgE assays are superior to conventional allergy tests for shrimp allergy diagnosis. J Allergy Clin Immunol Pract 9(1):236-244 e9. 10.1016/j.jaip.2020.08.057 32931950 10.1016/j.jaip.2020.08.057

[70] Yang AC, Arruda LK, Santos AB, Barbosa MC, Chapman MD, Galvao CE et al (2010) Measurement of IgE antibodies to shrimp tropomyosin is superior to skin prick testing with commercial extract and measurement of IgE to shrimp for predicting clinically relevant allergic reactions after shrimp ingestion. J Allergy Clin Immunol 125(4):872–878. 10.1016/j.jaci.2009.11.043 20226506 10.1016/j.jaci.2009.11.043

[71] Ukleja-Sokołowska N, Lis K, Żbikowska-Gotz M, Adamczak R, Kuźmiński A, Bartuzi Z (2021) Clinical utility of immunological methods based on the singleplex and multiplex ImmunoCap systems for diagnosis of shrimp allergy. J Int Med Res 49(4):03000605211006597. 10.1177/03000605211006597 33840250 10.1177/03000605211006597 PMC8044572

[72] Giovannini M, Beken B, Buyuktiryaki B, Barni S, Liccioli G, Sarti L et al (2023) IgE-mediated shellfish allergy in children. Nutrients 15:12. 10.3390/nu15122714 10.3390/nu15122714 PMC10301456 37375617

[73] Asero R, Mistrello G, Roncarolo D, Amato S (2002) A case of allergy to airborne, heat-labile shrimp allergens. J Allergy Clin Immunol 109(2):371–372. 10.1067/mai.2002.121311 11842314 10.1067/mai.2002.121311

[74] El-Qutob D (2017) Shrimp allergy: beyond avoidance diet. Eur Ann Allergy Clin Immunol 49(6):252–256. 10.23822/EurAnnACI.1764-1489.16 29249132 10.23822/EurAnnACI.1764-1489.16

[75] Morgan JE, O’Neil CE, Daul CB, Lehrer SB (1989) Species-specific shrimp allergens: RAST and RAST-inhibition studies. J Allergy Clin Immunol 83(6):1112–1117. 10.1016/0091-6749(89)90454-5 2732411 10.1016/0091-6749(89)90454-5

[76] Su BB, Blackmon W, Xu C, Holt C, Boateng N, Wang D et al (2024) Diagnosis and management of shrimp allergy. Front Allergy 5:1456999. 10.3389/falgy.2024.1456999 39493746 10.3389/falgy.2024.1456999 PMC11527777

[77] van Ree R, Antonicelli L, Akkerdaas JH, Garritani MS, Aalberse RC, Bonifazi F (1996) Possible induction of food allergy during mite immunotherapy. Allergy 51(2):108–113 8738516

[78] van Ree R, Antonicelli L, Akkerdaas JH, Pajno GB, Barberio G, Corbetta L et al (1996) Asthma after consumption of snails in house-dust-mite-allergic patients: a case of IgE cross-reactivity. Allergy 51(6):387–393 8837661

[79] Guilloux L, Vuitton DA, Delbourg M, Lagier A, Adessi B, Marchand CR et al (1998) Cross-reactivity between terrestrial snails (Helix species) and house-dust mite (Dermatophagoides pteronyssinus) II In vitro study. Allergy 53(2):151–158. 10.1111/j.1398-9995.1998.tb03863.x 9534913 10.1111/j.1398-9995.1998.tb03863.x

[80] Pajno GB, La Grutta S, Barberio G, Canonica GW, Passalacqua G (2002) Harmful effect of immunotherapy in children with combined snail and mite allergy. J Allergy Clin Immunol 109(4):627–629. 10.1067/mai.2002.122844 11941311 10.1067/mai.2002.122844

[81] Asero R (2005) Lack of de novo sensitization to tropomyosin in a group of mite-allergic patients treated by house dust mite-specific immunotherapy. Int Arch Allergy Immunol 137(1):62–65. 10.1159/000085105 15832051 10.1159/000085105

[82] Rossi RE, Monasterolo G, Incorvaia C, Moingeon P, Frati F, Passalacqua G et al (2010) Lack of neo-sensitization to Pen a 1 in patients treated with mite sublingual immunotherapy. Clin Mol Allergy 8:4. 10.1186/1476-7961-8-4 20230633 10.1186/1476-7961-8-4 PMC2859740

[83] Yang AC, Arruda LK, Santos ABR, Barbosa MCR, Chapman MD, Kalil J et al (2010) Cross-reactivity between mite and shrimp: the effect of immunotherapy with dust mite extract. J Allergy Clin Immunol 125(2):AB35. 10.1016/j.jaci.2009.12.168

[84] Cortellini G, Spadolini I, Santucci A, Cova V, Conti C, Corvetta A et al (2011) Improvement of shrimp allergy after sublingual immunotherapy for house dust mites: a case report. Eur Ann Allergy Clin Immunol 43(5):162–164 22145252

[85] Pevec B, Pevec MR, Markovic AS, Batista I (2014) House dust mite subcutaneous immunotherapy does not induce new sensitization to tropomyosin: does it do the opposite? J Investig Allergol Clin Immunol 24(1):29–34 24765878

[86] Farag-Mahmod FI, Hessam WI (2014) Life threatening shrimp allergy cross reacting with mite allergy : a case report. Allergy Asthma Clin Immunol 10(Suppl 1):A9. 10.1186/1710-1492-10-s1-a9

[87] Locke A, Hung L, Upton JEM, O’Mahony L, Hoang J, Eiwegger T (2023) An update on recent developments and highlights in food allergy. Allergy 78(9):2344–2360. 10.1111/all.15749 37087637 10.1111/all.15749

[88] Eiwegger T, Hung L, San Diego KE, O’Mahony L, Upton J (2019) Recent developments and highlights in food allergy. Allergy 74(12):2355–2367. 10.1111/all.14082 31593325 10.1111/all.14082

[89] Rachid R, Umetsu DT (2012) Immunological mechanisms for desensitization and tolerance in food allergy. Semin Immunopathol 34(5):689–702. 10.1007/s00281-012-0333-9 22821087 10.1007/s00281-012-0333-9 PMC3744633

[90] Ezhuthachan ID, Beaudoin M, Nowak-Wegrzyn A, Vickery BP (2024) The future of food allergy management: advancements in therapies. Curr Allergy Asthma Rep 24(4):161–171. 10.1007/s11882-024-01133-1 38393624 10.1007/s11882-024-01133-1

[91] Feuille E, Nowak-Wegrzyn A (2016) Oral immunotherapy for food allergies. Ann Nutr Metab 68(Suppl 1):19–31. 10.1159/000445391 27355816 10.1159/000445391

[92] Anagnostou A, Lieberman J, Greenhawt M, Mack DP, Santos AF, Venter C et al (2023) The future of food allergy: challenging existing paradigms of clinical practice. Allergy 78(7):1847–1865. 10.1111/all.15757 37129472 10.1111/all.15757

[93] Narisety SD, Keet CA (2012) Sublingual vs oral immunotherapy for food allergy: identifying the right approach. Drugs 72(15):1977–1989. 10.2165/11640800-000000000-00000 23009174 10.2165/11640800-000000000-00000 PMC3708591

[94] Andorf S, Purington N, Kumar D, Long A, O’Laughlin KL, Sicherer S et al (2019) A phase 2 randomized controlled multisite study using omalizumab-facilitated rapid desensitization to test continued vs discontinued dosing in multifood allergic individuals. EClinicalMedicine 7:27–38. 10.1016/j.eclinm.2018.12.006 31193674 10.1016/j.eclinm.2018.12.006 PMC6537534

[95] Pajno GB, Fernandez-Rivas M, Arasi S, Roberts G, Akdis CA, Alvaro-Lozano M et al (2018) EAACI guidelines on allergen immunotherapy: IgE-mediated food allergy. Allergy 73(4):799–815. 10.1111/all.13319 29205393 10.1111/all.13319

[96] Nguyen DI, Sindher SB, Chinthrajah RS, Nadeau K, Davis CM (2022) Shrimp-allergic patients in a multi-food oral immunotherapy trial. Pediatr Allergy Immunol 33(1):e13679. 10.1111/pai.13679 34655480 10.1111/pai.13679 PMC9297938

[97] Schoos AM, Chan ES, Wong T, Erdle SC, Chomyn A, Soller L et al (2024) Bypassing the build-up phase for oral immunotherapy in shrimp-allergic children. World Allergy Organ J 17(2):100865. 10.1016/j.waojou.2023.100865 38351903 10.1016/j.waojou.2023.100865 PMC10862060

[98] Fernandes A, Cao S, Parsons E, Bogetic D, Kumar D, Rogers J et al (2024) Clinical and mechanistic findings from MOTIF Trial: a phase 2 study using food allergen oral immunotherapy for cashew or shrimp allergy. J Allergy Clin Immunol 153(2):AB67. 10.1016/j.jaci.2023.11.230

[99] Fang L, Zhou F, Wu F, Yan Y, He Z, Yuan X et al (2021) A mouse allergic asthma model induced by shrimp tropomyosin. Int Immunopharmacol 91:107289. 10.1016/j.intimp.2020.107289 33370683 10.1016/j.intimp.2020.107289

[100] Nunes IV, Andrade CM, Guerra PV, Khouri MI, Galantini MPL, da Silva RAA et al (2023) A new experimental model to study shrimp allergy. Immunol Lett 260:73–80. 10.1016/j.imlet.2023.06.007 37315848 10.1016/j.imlet.2023.06.007

[101] Liu GM, Li B, Yu HL, Cao MJ, Cai QF, Lin JW et al (2012) Induction of mud crab (Scylla paramamosain) tropomyosin and arginine kinase specific hypersensitivity in BALB/c mice. J Sci Food Agric 92(2):232–238. 10.1002/jsfa.4565 21780134 10.1002/jsfa.4565

[102] Marhaeny HD, Pratama YA, Rohmah L, Kasatu SM, Miatmoko A, Khotib J (2023) Development of gastro-food allergy model in shrimp allergen extract-induced sensitized mice promotes mast cell degranulation. J Public Health Afr 14(Suppl 1):2512. 10.4081/jphia.2023.2512 37492545 10.4081/jphia.2023.2512 PMC10365647

[103] Ballmer-Weber BK, Fernandez-Rivas M, Beyer K, Defernez M, Sperrin M, Mackie AR et al (2015) How much is too much? Threshold dose distributions for 5 food allergens. J Allergy Clin Immunol 135(4):964–971. 10.1016/j.jaci.2014.10.047 25589011 10.1016/j.jaci.2014.10.047

[104] Houben GF, Baumert JL, Blom WM, Kruizinga AG, Meima MY, Remington BC et al (2020) Full range of population eliciting dose values for 14 priority allergenic foods and recommendations for use in risk characterization. Food Chem Toxicol 146:111831. 10.1016/j.fct.2020.111831 33166672 10.1016/j.fct.2020.111831 PMC7864389

[105] Nordlee JA, Remington BC, Ballmer-Weber BK, Lehrer SB, Baumert JL, Taylor SL (2013) Threshold dose for shrimp: a risk characterization based on objective reactions in clinical studies. J Allergy Clin Immunol 131(2):AB88. 10.1016/j.jaci.2012.12.980

[106] Leung NYH, Wai CYY, Shu SA, Chang CC, Chu KH, Leung PSC (2017) Low-dose allergen-specific immunotherapy induces tolerance in a murine model of shrimp allergy. Int Arch Allergy Immunol 174(2):86–96. 10.1159/000479694 29065408 10.1159/000479694

[107] Refaat M, El-Damhougy K, Sadiq A, Attia M, Mabrouk M (2013) Desensitization by sublingual immunotherapy for crustacean allergy. J Allergy Clin Immunol 131(2):AB88. 10.1016/j.jaci.2012.12.981

[108] Refaat MM, Attia MY, Saber HM (2014) Desensitization efficacy by sublingual immunotherapy of shrimps extract in asthmatic, rhinitis and urticaria allergic patients. Food Nutr Sci 5(17):1704–1710. 10.4236/fns.2014.517183

[109] Theodoropoulou LM, Cullen NA (2024) Sublingual immunotherapy for allergy to shrimp: the nine-year clinical experience of a Midwest Allergy-Immunology practice. Allergy Asthma Clin Immunol 20(1):33. 10.1186/s13223-024-00895-7 38734651 10.1186/s13223-024-00895-7 PMC11088126

[110] Zuidmeer-Jongejan L, Huber H, Swoboda I, Rigby N, Versteeg SA, Jensen BM et al (2015) Development of a hypoallergenic recombinant parvalbumin for first-in-man subcutaneous immunotherapy of fish allergy. Int Arch Allergy Immunol 166(1):41–51. 10.1159/000371657 25765512 10.1159/000371657

[111] Akdis CA, Akdis M, Boyd SD, Sampath V, Galli SJ, Nadeau KC (2023) Allergy: Mechanistic insights into new methods of prevention and therapy. Sci Transl Med 15(679):eadd2563. 10.1126/scitranslmed.add2563 36652536 10.1126/scitranslmed.add2563 PMC12998978

[112] Pan M, Yang J, Liu K, Xie X, Hong L, Wang S et al (2021) Irradiation technology: an effective and promising strategy for eliminating food allergens. Food Res Int 148:110578. 10.1016/j.foodres.2021.110578 34507726 10.1016/j.foodres.2021.110578

[113] Ayuso R, Sanchez-Garcia S, Pascal M, Lin J, Grishina G, Fu Z et al (2012) Is epitope recognition of shrimp allergens useful to predict clinical reactivity? Clin Exp Allergy 42(2):293–304. 10.1111/j.1365-2222.2011.03920.x 22192087 10.1111/j.1365-2222.2011.03920.x

[114] Willison LN, Zhang Q, Su M, Teuber SS, Sathe SK, Roux KH (2013) Conformational epitope mapping of Pru du 6, a major allergen from almond nut. Mol Immunol 55(3–4):253–263. 10.1016/j.molimm.2013.02.004 23498967 10.1016/j.molimm.2013.02.004

[115] Liu C, Sathe SK (2018) Food allergen epitope mapping. J Agric Food Chem 66(28):7238–7248. 10.1021/acs.jafc.8b01967 29924613 10.1021/acs.jafc.8b01967

[116] Li M, Xia F, Chen Y, Liu M, Liu Q, Yang Y et al (2022) Two hypo-allergenic derivatives lacking the dominant linear epitope of Scy p 1 and Scy p 3. Food Chem 373(Pt B):131588. 10.1016/j.foodchem.2021.131588 34801289 10.1016/j.foodchem.2021.131588

[117] Gazme B, Rezaei K, Udenigwe CC (2020) Effect of enzyme immobilization and in vitro digestion on the immune-reactivity and sequence of IgE epitopes in egg white proteins. Food Funct 11(7):6632–6642. 10.1039/d0fo00938e 32656552 10.1039/d0fo00938e

[118] Bavaro SL, Di Stasio L, Mamone G, De Angelis E, Nocerino R, Canani RB et al (2018) Effect of thermal/pressure processing and simulated human digestion on the immunoreactivity of extractable peanut allergens. Food Res Int 109:126–137. 10.1016/j.foodres.2018.04.021 29803434 10.1016/j.foodres.2018.04.021

[119] Sun N, Liu Y, Liu K, Wang S, Liu Q, Lin S (2022) Gastrointestinal fate of food allergens and its relationship with allergenicity. Compr Rev Food Sci Food Saf 21(4):3376–3404. 10.1111/1541-4337.12989 35751399 10.1111/1541-4337.12989

[120] Liu Y, Lin S, Liu K, Wang S, Sun N (2022) Gastrointestinal digestion products of shrimp (Penaeus vannamei) proteins retain an allergenic potential. Food Res Int 162(Pt A):111916. 10.1016/j.foodres.2022.111916 36461182 10.1016/j.foodres.2022.111916

[121] Huang YY, Liu GM, Cai QF, Weng WY, Maleki SJ, Su WJ et al (2010) Stability of major allergen tropomyosin and other food proteins of mud crab (Scylla serrata) by in vitro gastrointestinal digestion. Food Chem Toxicol 48(5):1196–1201. 10.1016/j.fct.2010.02.010 20156517 10.1016/j.fct.2010.02.010

[122] Guo Y, Li Z, Lin H (2009) The effect of simulated gastrointestinal digestion on shrimp Penaeus vannamei allergenicity. Chin J Oceanol Limnol 27(4):703–707. 10.1007/s00343-009-9190-3

[123] Liu GM, Cao MJ, Huang YY, Cai QF, Weng WY, Su WJ (2010) Comparative study of in vitro digestibility of major allergen tropomyosin and other food proteins of Chinese mitten crab (Eriocheir sinensis). J Sci Food Agric 90(10):1614–1620. 10.1002/jsfa.3988 20564455 10.1002/jsfa.3988

[124] Liu K, Lin S, Gao X, Wang S, Liu Y, Liu Q et al (2023) Reduced allergenicity of shrimp (Penaeus vannamei) by altering the protein fold, digestion susceptibility, and allergen epitopes. J Agric Food Chem 71(23):9120–9134. 10.1021/acs.jafc.3c01557 37257052 10.1021/acs.jafc.3c01557

[125] Zhao J, Li Y, Xu L, Ji Y, Zeng J, Timira V et al (2022) Insight into IgG/IgE binding ability, in vitro digestibility and structural changes of shrimp (Litopenaeus vannamei) soluble extracts with thermal processing. Food Chem 381:132177. 10.1016/j.foodchem.2022.132177 35121318 10.1016/j.foodchem.2022.132177

[126] Yadzir ZH, Misnan R, Bakhtiar F, Abdullah N, Murad S (2015) Tropomyosin, the major tropical oyster Crassostrea belcheri allergen and effect of cooking on its allergenicity. Allergy Asthma Clin Immunol 11:30. 10.1186/s13223-015-0099-4 26504467 10.1186/s13223-015-0099-4 PMC4620636

[127] Tong WS, Li S, Leung NYH, Wong WT, Leung TF, Leung PSC et al (2024) Shrimp extract exacerbates allergic immune responses in mice: implications on clinical diagnosis of shellfish allergy. Clin Rev Allergy Immunol 66(2):250–259. 10.1007/s12016-024-08994-4 38775874 10.1007/s12016-024-08994-4 PMC11193834

[128] Usui M, Harada A, Yasumoto S, Sugiura Y, Nishidai A, Ikarashi M et al (2015) Relationship between the risk for a shrimp allergy and freshness or cooking. Biosci Biotechnol Biochem 79(10):1698–1701. 10.1080/09168451.2015.1045830 25966963 10.1080/09168451.2015.1045830

[129] Fang L, Li G, Gu R, Cai M, Lu J (2018) Influence of thermal treatment on the characteristics of major oyster allergen Cra g 1 (tropomyosin). J Sci Food Agric 98(14):5322–5328. 10.1002/jsfa.9071 29656413 10.1002/jsfa.9071

[130] Abramovitch JB, Lopata AL, O’Hehir RE, Rolland JM (2017) Effect of thermal processing on T cell reactivity of shellfish allergens - discordance with IgE reactivity. PLoS One 12(3):e0173549. 10.1371/journal.pone.0173549 28273149 10.1371/journal.pone.0173549 PMC5342306

[131] Lasekan AO, Nayak B (2016) Effects of buffer additives and thermal processing methods on the solubility of shrimp (Penaeus monodon) proteins and the immunoreactivity of its major allergen. Food Chem 200:146–153. 10.1016/j.foodchem.2016.01.015 26830572 10.1016/j.foodchem.2016.01.015

[132] Zhenxing L, Hong L, Limin C, Jamil K (2007) Impact of irradiation and thermal processing on the immunoreactivity of shrimp (Penaeus vannamei) proteins. J Sci Food Agric 87(6):951–956. 10.1002/jsfa.2746

[133] Li Z-x, Hong L, Limin C (2006) Influence of ultrasonic treatment on the allergenic properties of shrimp (Penaeus vannamei) allergen. J Ocean Univ China 5:115–118

[134] Yu HL, Cao MJ, Cai QF, Weng WY, Su WJ, Liu GM (2011) Effects of different processing methods on digestibility of Scylla paramamosain allergen (tropomyosin). Food Chem Toxicol 49(4):791–798. 10.1016/j.fct.2010.11.046 21130825 10.1016/j.fct.2010.11.046

[135] Zhang Z, Li Z, Lin H (2021) Reducing the allergenicity of shrimp tropomyosin and allergy desensitization based on glycation modification. J Agric Food Chem 69(49):14742–14750. 10.1021/acs.jafc.1c03953 34427086 10.1021/acs.jafc.1c03953

[136] Zhang Z, Li XM, Li Z, Lin H (2021) Investigation of glycated shrimp tropomyosin as a hypoallergen for potential immunotherapy. Food Funct 12(6):2750–2759. 10.1039/d0fo03039b 33683237 10.1039/d0fo03039b

[137] Zhao J, Wang J, Xu L, Wang H, Zhang Z, Lin H et al (2023) Insights into the mechanism underlying the influence of glycation with different saccharides and temperatures on the IgG/IgE binding ability, immunodetection, in vitro digestibility of shrimp (Litopenaeus vannamei) tropomyosin. Foods 12(16):3049. 10.3390/foods12163049 37628047 10.3390/foods12163049 PMC10453262

[138] Zhang Z, Xiao H, Zhou P (2019) Allergenicity suppression of tropomyosin from Exopalaemon modestus by glycation with saccharides of different molecular sizes. Food Chem 288:268–275. 10.1016/j.foodchem.2019.03.019 30902292 10.1016/j.foodchem.2019.03.019

[139] Fu L, Wang C, Wang J, Ni S, Wang Y (2019) Maillard reaction with ribose, galacto-oligosaccharide or chitosan-oligosaccharide reduced the allergenicity of shrimp tropomyosin by inducing conformational changes. Food Chem 274:789–795. 10.1016/j.foodchem.2018.09.068 30373009 10.1016/j.foodchem.2018.09.068

[140] Han X, Wang X, Chen X, Liu H, Liu J, Waye MMY et al (2024) Intervention efficacy of slightly processed allergen/meat in oral immunotherapy for seafood allergy: a systematic review, meta-analysis, and meta-regression analysis in mouse models and clinical patients. Nutrients 16(5):667. 10.3390/nu16050667 38474795 10.3390/nu16050667 PMC10934674

[141] Wang Y, Ni S, Wang C, Li X, Fu L (2019) Cross-linking of shrimp tropomyosin catalyzed by transglutaminase and tyrosinase produces hypoallergens for potential immunotherapy. Food Funct 10(3):1609–1618. 10.1039/c9fo00046a 30820502 10.1039/c9fo00046a

[142] Yu S, Kuan YC, Chang CF, Liaw ET, Huang ES, Lin JF et al (2023) The effect of papain hydrolysis on tropomyosin levels in shrimp. Heliyon 9(12):e22410. 10.1016/j.heliyon.2023.e22410 38076185 10.1016/j.heliyon.2023.e22410 PMC10709369

[143] Zhang J, Liu W, Zhang R, Zhao X, Fang L, Qin X et al (2020) Hypoallergenic mutants of the major oyster allergen Cra g 1 alleviate oyster tropomyosin allergenic potency. Int J Biol Macromol 164:1973–1983. 10.1016/j.ijbiomac.2020.07.325 32758611 10.1016/j.ijbiomac.2020.07.325

[144] Chanasit S, Johnston E, Thanasarnthungcharoen C, Kamath SD, Bohle B, Lopata AL et al (2024) Hypoallergenic chimeric virus-like particles for the development of shrimp tropomyosin allergen Pen m 1-specific blocking antibodies. Allergy 79(4):1052–1055. 10.1111/all.15892 37753807 10.1111/all.15892

[145] Bachmann MF, Mohsen MO, Kramer MF, Heath MD (2020) Vaccination against allergy: a paradigm shift? Trends Mol Med 26(4):357–368. 10.1016/j.molmed.2020.01.007 32277930 10.1016/j.molmed.2020.01.007

[146] Wai CY, Leung NY, Ho MH, Gershwin LJ, Shu SA, Leung PS et al (2014) Immunization with hypoallergens of shrimp allergen tropomyosin inhibits shrimp tropomyosin specific IgE reactivity. PLoS On 9(11):e111649. 10.1371/journal.pone.0111649 10.1371/journal.pone.0111649 PMC4218792 25365343

[147] Myrset HR, Faeste CK, Kristiansen PE, Dooper MM (2013) Mapping of the immunodominant regions of shrimp tropomyosin Pan b 1 by human IgE-binding and IgE receptor crosslinking studies. Int Arch Allergy Immunol 162(1):25–38. 10.1159/000350791 23817275 10.1159/000350791

[148] Mou H, Gao M-x, Zhao J, Zhao X, Wang Z-d, Pan J-r (2014) Influence of gamma irradiation and heat treatment on the immunogenicity of five epitopes of Pen a1. Food Sci Technol Res 20(5):955–960. 10.3136/fstr.20.955

[149] Reese G, Viebranz J, Leong-Kee SM, Plante M, Lauer I, Randow S et al (2005) Reduced allergenic potency of VR9-1, a mutant of the major shrimp allergen Pen a 1 (Tropomyosin)1. J Immunol 175(12):8354–8364. 10.4049/jimmunol.175.12.8354 16339577 10.4049/jimmunol.175.12.8354

[150] Chen YX, He XR, Yang SQ, Huan F, Li DX, Yang Y et al (2023) IgE epitope analysis and hypo-immunoreactivity derivative of arginine kinase in mantis shrimp (Oratosquilla oratoria). J Agric Food Chem 71(24):9508–9518. 10.1021/acs.jafc.3c01549 37289596 10.1021/acs.jafc.3c01549

[151] Sin JI, Bagarazzi M, Pachuk C, Weiner DB (1999) DNA priming-protein boosting enhances both antigen-specific antibody and Th1-type cellular immune responses in a murine herpes simplex virus-2 gD vaccine model. DNA Cell Biol 18(10):771–779. 10.1089/104454999314917 10541436 10.1089/104454999314917

[152] Smeekens JM, Kesselring JR, Frizzell H, Bagley KC, Kulis MD (2022) Induction of food-specific IgG by Gene Gun-delivered DNA vaccines. Front Allergy 3:969337. 10.3389/falgy.2022.969337 36340020 10.3389/falgy.2022.969337 PMC9632862

[153] Rush CM, Mitchell TJ, Garside P (2010) A detailed characterisation of the distribution and presentation of DNA vaccine encoded antigen. Vaccine 28(6):1620–1634. 10.1016/j.vaccine.2009.11.014 20035828 10.1016/j.vaccine.2009.11.014 PMC2824851

[154] Wai CYY, Leung NYH, Leung PSC, Chu KH (2019) Modulating shrimp tropomyosin-mediated allergy: hypoallergen DNA vaccines induce regulatory T cells to reduce hypersensitivity in mouse model. Int J Mol Sci. 20(18):4656. 10.3390/ijms20184656 31546958 10.3390/ijms20184656 PMC6769673

[155] Kubo K, Takeda S, Uchida M, Maeda M, Endo N, Sugahara S et al (2022) Lit-LAMP-DNA-vaccine for shrimp allergy prevents anaphylactic symptoms in a murine model. Int Immunopharmacol 113(Pt A):109394. 10.1016/j.intimp.2022.109394 36334369 10.1016/j.intimp.2022.109394

[156] Valenta R, Campana R, Marth K, van Hage M (2012) Allergen-specific immunotherapy: from therapeutic vaccines to prophylactic approaches. J Intern Med 272(2):144–157. 10.1111/j.1365-2796.2012.02556.x 22640224 10.1111/j.1365-2796.2012.02556.x PMC4573524

[157] Reche PA, Fernandez-Caldas E, Flower DR, Fridkis-Hareli M, Hoshino Y (2014) Peptide-based immunotherapeutics and vaccines. J Immunol Res 2014:256784. 10.1155/2014/256784 24741581 10.1155/2014/256784 PMC3987874

[158] El-Qutob D, Reche P, Subiza JL, Fernandez-Caldas E (2015) Peptide-based allergen specific immunotherapy for the treatment of allergic disorders. Recent Pat Inflamm Allergy Drug Discov 9(1):16–22. 10.2174/1872213x09666150302105555 25760734 10.2174/1872213x09666150302105555

[159] Larche M (2007) Immunotherapy with allergen peptides. Allergy Asthma Clin Immunol 3(2):53–59. 10.1186/1710-1492-3-2-53 20525144 10.1186/1710-1492-3-2-53 PMC2873623

[160] Ravkov EV, Pavlov IY, Martins TB, Gleich GJ, Wagner LA, Hill HR et al (2013) Identification and validation of shrimp-tropomyosin specific CD4 T cell epitopes. Hum Immunol 74(12):1542–1549. 10.1016/j.humimm.2013.08.276 23993987 10.1016/j.humimm.2013.08.276 PMC3870591

[161] Wai CY, Leung NY, Leung PS, Chu KH (2016) T cell epitope immunotherapy ameliorates allergic responses in a murine model of shrimp allergy. Clin Exp Allergy 46(3):491–503. 10.1111/cea.12684 26610061 10.1111/cea.12684

[162] Gao Q, Hong J, Xiao X, Cao H, Yuan R, Liu Z et al (2020) T cell epitope of arginine kinase with CpG co-encapsulated nanoparticles attenuates a shrimp allergen-induced Th2-bias food allergy. Biosci Biotechnol Biochem 84(4):804–814. 10.1080/09168451.2019.1699395 31795812 10.1080/09168451.2019.1699395

[163] Leung NY, Wai CY, Ho MH, Liu R, Lam KS, Wang JJ et al (2017) Screening and identification of mimotopes of the major shrimp allergen tropomyosin using one-bead-one-compound peptide libraries. Cell Mol Immunol 14(3):308–318. 10.1038/cmi.2015.83 26364917 10.1038/cmi.2015.83 PMC5360880

[164] Dantzer JA, Wood RA (2025) Anti-IgE and food allergy. J Allergy Clin Immunol 155(1):1–11. 10.1016/j.jaci.2024.10.020 39505277 10.1016/j.jaci.2024.10.020

[165] Zuberbier T, Wood RA, Bindslev-Jensen C, Fiocchi A, Chinthrajah RS, Worm M et al (2023) Omalizumab in IgE-mediated food allergy: a systematic review and meta-analysis. J Allergy Clin Immunol Pract 11(4):1134–1146. 10.1016/j.jaip.2022.11.036 36529441 10.1016/j.jaip.2022.11.036

[166] Leung DY, Sampson HA, Yunginger JW, Burks AW Jr, Schneider LC, Wortel CH et al (2003) Effect of anti-IgE therapy in patients with peanut allergy. N Engl J Med 348(11):986–993. 10.1056/NEJMoa022613 12637608 10.1056/NEJMoa022613

[167] Gasser P, Tarchevskaya SS, Guntern P, Brigger D, Ruppli R, Zbaren N et al (2020) The mechanistic and functional profile of the therapeutic anti-IgE antibody ligelizumab differs from omalizumab. Nat Commun 11(1):165. 10.1038/s41467-019-13815-w 31913280 10.1038/s41467-019-13815-w PMC6949303

[168] Benito-Villalvilla C, de la Rocha-Munoz A, Lopez-Abente J, Eggel A, Bottoli I, Severin T et al (2023) Ligelizumab impairs IgE-binding to plasmacytoid dendritic cells more potently than omalizumab and restores IFN-alpha production and FOXP3(+) Treg generation. Allergy 78(4):1060–1072. 10.1111/all.15567 36315052 10.1111/all.15567

[169] Wood RA, Togias A, Sicherer SH, Shreffler WG, Kim EH, Jones SM et al (2024) Omalizumab for the treatment of multiple food allergies. N Engl J Med 390(10):889–899. 10.1056/NEJMoa2312382 38407394 10.1056/NEJMoa2312382 PMC11193494

[170] Rafi A, Do LT, Katz R, Sheinkopf LE, Simons CW, Klaustermeyer W (2010) Effects of omalizumab in patients with food allergy. Allergy Asthma Proc 31(1):76–83. 10.2500/aap.2010.31.3304 20167148 10.2500/aap.2010.31.3304

[171] Diesner SC, Bergmayr C, Pfitzner B, Assmann V, Krishnamurthy D, Starkl P et al (2016) A distinct microbiota composition is associated with protection from food allergy in an oral mouse immunization model. Clin Immunol 173:10–18. 10.1016/j.clim.2016.10.009 27789346 10.1016/j.clim.2016.10.009 PMC5464391

[172] Wang X, Zhang P, Zhang X (2021) Probiotics regulate gut microbiota: an effective method to improve immunity. Molecules 26(19):6076. 10.3390/molecules26196076 34641619 10.3390/molecules26196076 PMC8512487

[173] Liu Y, Wang J, Wu C (2021) Modulation of gut microbiota and immune system by probiotics, pre-biotics, and post-biotics. Front Nutr 8:634897. 10.3389/fnut.2021.634897 35047537 10.3389/fnut.2021.634897 PMC8761849

[174] Pratap K, Taki AC, Johnston EB, Lopata AL, Kamath SD (2020) A comprehensive review on natural bioactive compounds and probiotics as potential therapeutics in food allergy treatment. Front Immunol 11:996. 10.3389/fimmu.2020.00996 32670266 10.3389/fimmu.2020.00996 PMC7326084

[175] Schiavi E, Barletta B, Butteroni C, Corinti S, Boirivant M, Di Felice G (2011) Oral therapeutic administration of a probiotic mixture suppresses established Th2 responses and systemic anaphylaxis in a murine model of food allergy. Allergy 66(4):499–508. 10.1111/j.1398-9995.2010.02501.x 21058959 10.1111/j.1398-9995.2010.02501.x

[176] Fu L, Fu S, Wang C, Xie M, Wang Y (2019) Yogurt-sourced probiotic bacteria alleviate shrimp tropomyosin-induced allergic mucosal disorders, potentially through microbiota and metabolism modifications. Allergol Int 68(4):506–514. 10.1016/j.alit.2019.05.013 31262632 10.1016/j.alit.2019.05.013

[177] Fu L, Song J, Wang C, Fu S, Wang Y (2017) Bifidobacterium infantis potentially alleviates shrimp tropomyosin-induced allergy by tolerogenic dendritic cell-dependent induction of regulatory T cells and alterations in gut microbiota. Front Immunol 8:1536. 10.3389/fimmu.2017.01536 29176981 10.3389/fimmu.2017.01536 PMC5686061

[178] Wang HT, Anvari S, Anagnostou K (2019) The role of probiotics in preventing allergic disease. Children 6(2):. 10.3390/children6020024 10.3390/children6020024 PMC6406271 30764558

[179] Wei Choo CY, Yeh KW, Huang JL, Su KW, Tsai MH, Hua MC et al (2021) Oxidative stress is associated with atopic indices in relation to childhood rhinitis and asthma. J Microbiol Immunol Infect 54(3):466–473. 10.1016/j.jmii.2020.01.009 32094074 10.1016/j.jmii.2020.01.009

[180] Li XM (2011) Treatment of asthma and food allergy with herbal interventions from traditional Chinese medicine. Mt Sinai J Med 78(5):697–716. 10.1002/msj.20294 21913200 10.1002/msj.20294 PMC4118473

[181] Li XM, Zhang TF, Huang CK, Srivastava K, Teper AA, Zhang L et al (2001) Food Allergy Herbal Formula-1 (FAHF-1) blocks peanut-induced anaphylaxis in a murine model. J Allergy Clin Immunol 108(4):639–646. 10.1067/mai.2001.118787 11590394 10.1067/mai.2001.118787

[182] Wang J, Jones SM, Pongracic JA, Song Y, Yang N, Sicherer SH et al (2015) Safety, clinical, and immunologic efficacy of a Chinese herbal medicine (Food Allergy Herbal Formula-2) for food allergy. J Allergy Clin Immunol 136(4):962–970. 10.1016/j.jaci.2015.04.029 26044855 10.1016/j.jaci.2015.04.029 PMC4600418

[183] Patil SP, Wang J, Song Y, Noone S, Yang N, Wallenstein S et al (2011) Clinical safety of Food Allergy Herbal Formula-2 (FAHF-2) and inhibitory effect on basophils from patients with food allergy: extended phase I study. J Allergy Clin Immunol 128(6):1259–1652. 10.1016/j.jaci.2011.06.015 21794906 10.1016/j.jaci.2011.06.015 PMC3229682

[184] Wang J, Patil SP, Yang N, Ko J, Lee J, Noone S et al (2010) Safety, tolerability, and immunologic effects of a food allergy herbal formula in food allergic individuals: a randomized, double-blinded, placebo-controlled, dose escalation, phase 1 study. Ann Allergy Asthma Immunol 105(1):75–84. 10.1016/j.anai.2010.05.005 20642207 10.1016/j.anai.2010.05.005 PMC3026589

[185] Srivastava KD, Sampson HA, Li X (2009) The traditional Chinese medicine formula FAHF-2 provides complete protection from anaphylaxis in a murine model of multiple food allergy. J Allergy Clin Immunol 123(2):S151. 10.1016/j.jaci.2008.12.565

[186] Terwee CB (2008) Succesful treatment of food allergy with Nambudripad’s Allergy Elimination Techniques (NAET) in a 3-year old: a case report. Cases J 1(1):166. 10.1186/1757-1626-1-166 18803817 10.1186/1757-1626-1-166 PMC2556663

[187] Sindher SB, Kumar D, Cao S, Purington N, Long A, Sampath V et al (2022) Phase 2, randomized multi oral immunotherapy with omalizumab ‘real life’ study. Allergy 77(6):1873–1884. 10.1111/all.15217 35014049 10.1111/all.15217

[188] Piscia-Nichols C, Levin-Young O, Kirchner J, Sullivan A (2024) A patient-centric approach in the harmony phase 1/2 trial of ADP101 for food allergies: incorporating the patient voice. J of Allergy Clin Immunol 153(2):AB120. 10.1016/j.jaci.2023.11.398

